# Intercellular Adhesion Molecule-1 (ICAM-1) and ICAM-2 Differentially Contribute to Peripheral Activation and CNS Entry of Autoaggressive Th1 and Th17 Cells in Experimental Autoimmune Encephalomyelitis

**DOI:** 10.3389/fimmu.2019.03056

**Published:** 2020-01-14

**Authors:** Neda Haghayegh Jahromi, Luca Marchetti, Federica Moalli, Donovan Duc, Camilla Basso, Heidi Tardent, Elisa Kaba, Urban Deutsch, Caroline Pot, Federica Sallusto, Jens V. Stein, Britta Engelhardt

**Affiliations:** ^1^Theodor Kocher Institute, University of Bern, Bern, Switzerland; ^2^Laboratories of Neuroimmunology, Division of Neurology and Neuroscience Research Center, Department of Clinical Neurosciences, Lausanne University Hospital, University of Lausanne, Epalinges, Switzerland; ^3^Institute for Research in Biomedicine, Università della Svizzera Italiana, Bellinzona, Switzerland; ^4^Department of Biology, Institute of Microbiology, ETH Zurich, Zurich, Switzerland

**Keywords:** experimental autoimmune encephalomyelitis, ICAM-1, ICAM-2, dendritic cells, Th1 cells, Th17 cells, blood-brain barrier, T cell activation

## Abstract

In experimental autoimmune encephalomyelitis (EAE), an animal model of multiple sclerosis (MS), myelin-specific T cells are activated in the periphery and differentiate in T helper (Th) 1 and Th17 effector cells, which cross the blood-brain barrier (BBB) to reach the central nervous system (CNS), where they induce neuroinflammation. Here, we explored the role of intercellular adhesion molecule-1 (ICAM-1) and ICAM-2 in the activation of naïve myelin-specific T cells and in the subsequent migration of differentiated encephalitogenic Th1 and Th17 cells across the BBB *in vitro* and *in vivo*. While on antigen-presenting cells ICAM-1, but not ICAM-2 was required for the activation of naïve CD4^+^ T cells, endothelial ICAM-1 and ICAM-2 mediated both Th1 and Th17 cell migration across the BBB. ICAM-1/-2-deficient mice developed ameliorated typical and atypical EAE transferred by encephalitogenic Th1 and Th17 cells, respectively. Our study underscores important yet cell-specific contributions for ICAM-1 and ICAM-2 in EAE pathogenesis.

## Introduction

Multiple sclerosis (MS) is considered an autoimmune inflammatory demyelinating disease of the central nervous system (CNS). Experimental autoimmune encephalomyelitis (EAE), a prototypic animal model for MS, mimics many aspects of the acute inflammatory phase of the human disease (1). In EAE, naïve myelin-reactive CD4^+^ T cells are activated and differentiated in peripheral lymphoid tissue into encephalitogenic Th1 or Th17 cells, which travel in the blood circulation to the CNS. After crossing the blood-brain barrier (BBB) they next infiltrate in the CNS parenchyma, leading to clinical manifestation of the disease (2). EAE can be actively induced by immunization with CNS myelin antigens emulsified in complete Freund's adjuvant (aEAE) or by injection of myelin-reactive CD4^+^ T cells into syngeneic naïve recipients (tEAE) (3, 4).

Activation of naïve CD4^+^ T cells during aEAE occurs in the draining peripheral lymph nodes (dLNs), where T cells recognize their cognate antigen (Ag) on antigen-presenting cells (APCs) forming periodic contacts between the T-cell receptor (TCR) and the myelin oligodendrocyte glycoprotein (MOG)_aa35−55_ peptide loaded major histocompatibility complex (pMHC) on the APCs, referred to as the immunological synapse (IS) (5, 6). The fate of naïve T cells is determined within hours after Ag exposure by interacting with APCs in LN. The interaction between the integrin lymphocyte function associated antigen-1 (LFA-1) on the T cells and its ligand intercellular adhesion molecule-1 (ICAM-1) on the APCs is suggested to be involved in modulating the IS (7). However, APCs additionally express ICAM-2, an alternate ligand of LFA-1. While a role for ICAM-1 in IS formation and subsequent activation of naïve T cells is shown (7–9), the role of ICAM-2 on APCs in activation of autoaggressive T cells has not been addressed.

Activation of myelin specific CD4^+^ T cells is followed by their proliferation and differentiation into encephalitogenic T-cell subsets, namely IFN-γ producing T helper (Th) 1 and IL-17 producing Th17 cells (10, 11).

The effector phase of EAE pathogenesis starts with the migration of encephalitogenic Th1 and Th17 cells across the BBB. T-cell migration across the BBB is a multistep process mediated by the sequential interaction of adhesion and signaling molecules on the T cells and BBB endothelial cells (12). Hereby the migration of CD4^+^ T cells across the BBB is independent of their TCR specificity and rather depends on their activation status (13–15). Interaction of LFA-1 on the T cells with its vascular ligands ICAM-1 and ICAM-2 is essential for polarization and subsequent crawling of encephalitogenic Th1 cells on the BBB (14, 16). Recent observations suggested, however, that Th17 cells rather than Th1 cells rely on LFA-1 for CNS entry (17). Thus, the precise involvement of endothelial ICAM-1 and ICAM-2 in mediating Th1 vs. Th17-cell migration across the BBB in neuroinflammation remains to be explored.

In the present study we therefore investigated the involvement of ICAM-1 and ICAM-2 on antigen presenting cells and on the BBB endothelium in EAE pathogenesis.

## Materials and Methods

### Mice

C57BL/6J mice were obtained from Janvier (Genest Saint Isle, France). ICAM-1^null^ (Icam1^tm1Alb^) mice, generated by deletion of the entire coding region of the ICAM-1 gene (18), were kindly provided by Dr. D.C. Bullard (Birmingham, Alabama, USA). ICAM-2^−/−^ (Icam2^tm1Jcgr^) mice were described previously (19). ICAM-1^null^//ICAM-2^−/−^ double knockout mice were generated by breeding ICAM-1^null^ and ICAM-2^−/−^ mice (14). For the sake of readability, ICAM-1^null^, ICAM-2^−/−^, and ICAM-1^null^//ICAM-2^−/−^ mice are referred in the text as ICAM-1^−/−^, ICAM-2^−/−^, and ICAM-1/-2^−/−^ mice, respectively. 2D2 TCR MOG transgenic C57BL/6J mice (2D2) expressing a T-cell receptor recognizing MOG_aa35−55_ in the context of MHC class II (I-A^b^) were obtained from Dr. V.K. Kuchroo (Boston, USA) (20). 2D2 mice were crossed to ICAM-1^null^//ICAM-2^−/−^ and GFP-Tg [Tg(CAG-GFP)10Osb] (21) mice, respectively, to create 2D2 ICAM-1^null^//ICAM-2^−/−^ mice and 2D2 GFP mice. 2D2 Ubi-GFP mice were generated by breeding 2D2 mice with Ubi-GFP mice (22) [(B6J-Tg(Tcra2D2, Tcrb2D2)1Kuch; Tg(UBC-GFP)30Scha) (MGI ID 3700794; 3057178)]. 2D2 tdTomato mice were generated by breeding 2D2 mice with tdTomato mice [Gt(ROSA)^26Sortm14.1(CAG−tdTomato)Hze^]. tdTomato mice were generated by breeding Ai14 mice (23) with ZP3-Cre mice (24) [Gt(ROSA)26Sor^tm14(CAG−tdTomato)Hze^; Tg(Zp3-cre)93Knw] (MGI ID: 3809524; 2176187). 2D2 Ubi-GFP and Ai14 mice were obtained from the Swiss Immunological Mouse Repository (SwImMR). ZP3-Cre mice were kindly provided by Dr. Pawel Pelczar. All mouse lines were backcrossed to a C57BL/6J background for at least eight generations and all alleles were bred to homozygosity. Mice were housed in individually ventilated cages under specific pathogen-free conditions. Animal procedures were performed in accordance with the Swiss legislation on the protection of animals and were approved by the veterinary office of the Kanton of Bern.

### Flow Cytometry Antibodies

The following rat anti-mouse mAbs were used for flow cytometry: anti-CD3 (clone: 17A2, BioLegend), anti-CD4 (clone: RM4-5, BD Pharmingen), anti-CD11b (clone: M1/70, BioLegend), anti-CD11c (clone: HL3, BD Biosciences), anti-CD18 (clone: M18/2, Biolegend), anti-CD25 (clone: 3C7, BioLegend), anti-CD28 (clone: 37.51, BioLegend), anti-CD29 (clone: HMβ1-1, BioLegend), anti-CD44 (clone: IM7, BioLegend), anti-CD45 (clone: 30F11, BioLegend), anti-CD49d (clone: PS/2, Southern Biotech), anti-CD54 (clone: INY1/1.7, BioLegend), anti-CD69 (clone: H1.2F3, BioLegend), anti-CD80 (clone: 16-10A1, BioLegend), anti-CD86 (clone: GL1, BioLegend), anti-GM-CSF (clone: MP1-22E9, BioLegend), anti-IFN-γ (clone XMG1.2, eBioscience), anti-IL-4 (clone: 11B11, BioLegend), anti-IL-5 (clone: TRFK5, eBioscience), anti-IL-10 (clone: JES5-16E3, eBioscience), anti-IL-13 (clone: eBio13A, eBioscience), anti-IL-17A (clone: TC11-18H10, BD Bioscience), anti-MHCII (clone: 2G9, BD Biosciences), anti-RORγt (clone: AFKJS-9, eBioscience). The following mAbs were produced in our lab from hybridomas: anti-CD16/32 (clone: 2.4G2), anti-ICAM-1 (clone: 25ZC7), anti-ICAM-2 (clone: 3C4), anti-α4β7 (clone: DATK 32), anti-LFA-1 (clone: FD448.1), anti-PSGL-1 (clone: 4RA10), anti-α4 (clone: PS/2).

### Cytokines and Antibodies

Recombinant murine IL-1β and IL-12 were purchased from PeproTech (Rocky Hill, NJ, USA) and recombinant mouse IL-23, IL-1α, IL-6, and TGFβ1 were purchased from R&D Systems (Minneapolis, MN, USA). Recombinant IL-2 supernatant was purified from an in-house hybridoma. Anti-mouse CD3 (clone 145-2C11) and CD28 (clone 37.51) monoclonal antibodies (mAbs) were bought from Pharmingen BD Biosciences (Switzerland). Rat anti-IFN-γ mAb produced from the hybridoma cell line XMG1.2 was kindly provided by Jean-Charles Guery (Toulouse, France) (25). Anti-IL-4 mAb produced from the hybridoma cell line 11B11 was kindly provided by Dr. Benjamin Segal (Ohio, USA).

Rat-anti-mouse endoglin antibody (clone MJ7/18) (26) was purified endotoxin-free from hybridoma culture supernatants as previously described (27). Anti-mouse/human α4 integrin (clone PS/2) was bought from Bio X Cell (West Lebanon, NH, USA).

### DC Culture and Peptide Pulsing

Bone marrow derived dendritic cells (BM-derived DCs) culture was described before (28). Briefly, BM cell suspensions from WT, ICAM-1^−/−^, ICAM-2^−/−^, and ICAM-1/-2^−/−^ C57BL/6J mice were obtained by centrifugation (4,000 rpm, 4 min) of femurs and tibiae. BM cells were incubated, in 20 ml cultures containing 18 ml restimulation medium containing RPMI-1640 supplemented with 10% FBS (Thermo Fisher Scientific), 10 U/ml penicillin-streptomycin, 2 mM L-glutamine, 1% (v/v) non-essential amino acids, 1 mM sodium pyruvate, and 0.05 mM β-mercaptoethanol (Grogg Chemie AG) and 2 ml Flt-3L-containing supernatant [produced from SP2/0 transfected cell line secreting murine recombinant Flt-3L (29, 30)], for 6–9 days until activation with 1 μg/ml lipopolysaccharide (LPS from Salmonella; Sigma-Aldrich) for 24 h and pulsing with MOG_aa35−55_ peptide for 1 h at 37°C.

### Two-Photon Microscopy (2P-IVM) of Popliteal LNs

2P-IVM of popliteal LNs was done as described previously (28). Briefly, LPS-activated DCs from WT and ICAM-1/-2^−/−^ C57BL/6J mice were pulsed with 100 μg/ml of MOG_aa35−55_ peptide and fluorescently labeled with 2.5 μM CellTracker Red CMTMR (5-(and-6)-(((4-chloromethyl)benzoyl) amino)tetramethylrhodamine) or 20 μM CellTracker Blue CMAC (7-amino-4-chloromethylcoumarin) at 37°C for 20 min, with dyes swapped between experiments. After washing, a total of 4 × 10^6^ DCs consisting of a 1:1 mixture of WT and ICAM-1/-2^−/−^ labeled DCs were injected s.c. in right hindfoot of recipient C57BL/6J mice and CD4^+^ T cell transfer was performed 18 h later. Purified 2D2 GFP-expressing T cells (5 × 10^6^ /mouse) were injected i.v. into sex-matched 5–10 weeks old anesthetized recipient C57BL/6J mice surgically prepared to expose the right popliteal LN. Immediately after T cell transfer, 2P-IVM imaging was performed using the TrimScope system equipped with an Olympus BX50WI fluorescence microscope and a 20 × objective (NA 0.95; LaVision Biotec). 11–16 z-stacks (spacing 4 μm) of 250–300 × 250–300 μm x–y sections were acquired every 20 s for 20–30 min. Imaging was performed in the T cell area identified by the presence of HEVs labeled with Alexa Fluor 633–conjugated MECA-79 (15 μg/mouse). Sequences of image stacks were transformed into volume-rendered four-dimensional movies using Volocity software, which was also used for semi-automated tracking of cell motility in three dimensions. The average track speed was calculated from the x, y, and z coordinates of cell centroids.

### Analysis of T Cell Activation Markers and *in vivo* Proliferation

DCs isolated from WT and ICAM-1/-2^−/−^ C57BL/6J mice were stimulated *in vitro* and maturated with LPS. Mature WT and ICAM-1/-2^−/−^ DCs were pulsed *ex vivo* either with 2, 100 μg/ml, or no (control) MOG_aa35−55_ peptide. These two concentrations, 2 and 100 μg/ml MOG_aa35−55_ peptide, were selected as low and high concentrations of peptide based on our *in vitro* results of T-cell proliferation in the presence of various concentrations of MOG_aa35−55_ peptide. Each individual WT recipient C57BL/6J mouse was subcutaneously (s.c.) injected with 2 × 10^6^ Ag (low or high concentration) loaded ICAM-1/-2^−/−^ DCs into the right front and hind paw and with 2 × 10^6^ Ag (low or high concentration) loaded WT DCs into the left front and hind paw. As a control condition, other WT recipient C57BL/6J mice were s.c. injected with 2 × 10^6^ non-Ag loaded ICAM-1/-2^−/−^ DCs into the right front and hind paw and with 2 × 10^6^ non-Ag loaded WT DCs into the left front and hind paw. Naïve CD4^+^ T cells were harvested from the spleen and peripheral LNs of 2D2 GFP mice and the purity of CD4^+^ T cells was assessed by flow cytometry (Supplementary Figure 1A). 18 h after injection of pulsed DCs, naïve 2D2 CD4^+^ T cells expressing GFP were injected intravenously (i.v.) (5 × 10^6^/mouse) into the WT recipient C57BL/6J mice. 48 and 72 h after injection of naïve 2D2 GFP CD4^+^ T cells and homing to the LNs, T-cell activation was determined by flow cytometry analysis in LNs. At indicated time points, expression of CD25 and CD69 on transferred CD4^+^ T cells was measured by flow cytometry. For tracking T-cell proliferation, purified CD4^+^ T cells were labeled with the cell proliferation dye eFluor 670 (e670) (eBioscience) and injected into the recipient mice containing WT or ICAM-1/-2^−/−^ DCs. Recipients were sacrificed at 48 and 72 h after injection of naïve 2D2 GFP CD4^+^ T cells and single cell suspensions from brachial and popliteal LNs were prepared. Cells were stained for CD25, CD69 and CD4 and analyzed with an LSRII or FACSCalibur flow cytometer (BD). Diva software or CellQuest were used for data acquisition, FlowJo software (Version 10) was used for data analysis.

### Flow Cytometry Surface Staining of T Cells and DCs

Cells were stained with appropriate combinations of fluorophore-conjugated mAbs at saturating concentrations on ice in the dark for 30 min. Flow cytometry was performed using FACSCalibur with CellQuest software (BD Biosciences) or Attune NxT with Attune NxT Flow Cytometer software (Thermo Fisher Scientific) and analysis was done with FlowJo software (Version 10).

### *In vitro* T-Cell Proliferation

For splenic APCs, single cell suspension was prepared from harvested spleen of WT, ICAM-1^−/−^, ICAM-2^−/−^, and ICAM-1/-2^−/−^ C57BL/6J mice. Erythrocytes were depleted using freshly prepared lysis buffer [a mixture of nine volumes ACT I (155 mM NH_4_Cl) and 1 volume ACT II (170 mM Tris-HCL, pH 7.65)] at 37°C for 4 min. The resulting cell suspension was filtered through a sterile 100 μm nylon mesh and sub lethally irradiated (40 Gy).

Splenic APCs and LPS-matured DCs from WT, ICAM-1^−/−^, ICAM-2^−/−^, and ICAM-1/-2^−/−^ C57BL/6J mice were co cultured with purified CD4^+^ T cells harvested from 2D2 C57BL/6J mice for 72 h. To study the role of ICAM-1, ICAM-2 and both ICAM-1 and ICAM-2 on T cells, CD4^+^ T cells were harvested from spleens and pLNs of 2D2, 2D2 ICAM-1^−/−^, 2D2 ICAM-2^−/−^, and 2D2 ICAM-1/-2^−/−^ C57BL/6J mice purified via negative selection with magnetic beads (Dynal Invitrogen, Oslo, Norway) and co-cultured with irradiated APCs or DCs harvested from WT C57BL/6J mice. 5 × 10^5^ APCs with a ratio of 5:1 APC/T cell and 1 × 10^4^ DCs with a ratio of 1:10 DC/T cell were seeded per well in restimulation medium before MOG_aa35−55_ peptide was added. T-cell proliferation induced by cross-linking of CD3 and CD28 with 0.1 μg/ml of the respective antibodies was used as a positive control. T-cell proliferation in medium in the absence of antigen served as negative control. All samples were plated as triplicates. [^3^H] Thymidine ([^3^H]dT, 1 μCi/ml) was added 16 h before harvesting the cultures on glass-fiber filters using a cell harvester (Inotech, Dottikon, Switzerland). Filters were dissolved in 2 ml scintillation fluid and the incorporation of [^3^H]dT was measured by liquid scintillation counting as count per minute (cpm) (31, 32).

### *In vitro* Blood–Brain Barrier Model

Primary mouse brain microvascular endothelial cells (pMBMECs) were isolated from 7 to 9 weeks old WT or ICAM-1/-2^−/−^ C57BL/6J mice and cultured exactly as described before (16, 33). Where indicated, cytokine stimulation was performed for 16–20 h prior to the experiment with 10 ng/ml TNF-α or 20 ng/ml IL-1β.

### *In vitro* Polarization of Th1 and Th17 Cells for *in vitro* and *in vivo* Imaging

Naïve T cells were isolated from pLNs and spleen of 8–10 weeks old 2D2 mice. Spleens and pLNs were homogenized and filtered through a sterile 100 μm nylon mesh to prepare a single cell suspension. After erythrocyte lysis, CD4^+^ T cells were purified via negative selection using magnetic beads (Dynal Invitrogen, Oslo, Norway/EasySep™ mouse CD4^+^ T cell isolation kit, STEMCELL Technologies, Canada) according to the manufacturer's instructions. For helper T cell differentiation, naïve T cells were stimulated for 4 days with plate-bound antibody to CD3 (clone 145-2C11, 2 μg/ml, BioLegend) and CD28 (clone 37.51, 2 μg/ml, BioLegend). The naïve cells were cultured at a concentration of 1 × 10^5^/ml in restimulation medium containing RPMI-1640 supplemented with 10% FBS (Thermo Fisher Scientific), 10 U/ml penicillin-streptomycin, 2 mM L-glutamine, 1% (v/v) non-essential amino acids, 1 mM sodium pyruvate, and 0.05 mM β-mercaptoethanol (Grogg Chemie AG). Th1 cells were generated by addition of recombinant mouse IL-12 (5 ng/ml) into the culture medium. For the generation of Th17 cells, naïve T cells were cultured with recombinant mouse IL-6 (20 ng/ml), human TGF-β1 (1 ng/ml), and murine IL-1β (10 ng/ml). On day 4, all the cells were collected from the wells and transferred to Petri dishes at a concentration of 3 × 10^6^/ml using fresh restimulation medium containing 1% IL-2 cell supernatant (for Th1 cells) or containing 10 ng/ml IL-23 (for Th17 cells) and incubated at 37°C in 7% CO_2_ for additional 24 h. On day 5, freshly activated live cells were isolated by Nycoprep 1.077 A (Axis-Shield, Dundee, UK) density gradient centrifugation. For *in vitro* live cell imaging experiments, naïve T cells were isolated from 8 to 10 weeks old 2D2 Ubi-GFP or 2D2 tdTomato mice and cultured as above. Signature cytokine profile and trafficking phenotype of *in vitro* polarized Th1 and Th17 cells was assessed by flow cytometry (Supplementary Figures 4A,B).

### Cytokine Production Quantification by Flow Cytometry

Intracellular cytokine staining of T cells was performed after stimulation with 50 ng/ml phorbol myrisate acetate (PMA) (Enzo Life Sciences AG), 1 μg/ml Ionomycin (Enzo Life Sciences AG), and 6.6 μl/10 ml Golgi stop (BD Biosciences) for 4 h before harvesting the T cells. T cells were then fixed with Cytofix buffer (BD Biosciences) for 15 min at 20°C, washed with PBS, permeabilized with perm/wash buffer (BD Biosciences) for 15 min at 20°C and stained with fluorophore-conjugated mAbs and the respective isotype control mAbs. Flow cytometry was performed on a FACSCalibur using CellQuest software (BD Biosciences) or Attune NxT using Attune NxT Flow Cytometer software (Thermo Fisher Scientific) and analysis was done with FlowJo software (Version 10).

### *In vitro* Live Cell Imaging

*In vitro* live cell imaging of activated Th1 and Th17-cell extravasation across pMBMECs was performed as previously described (15, 16, 34). GFP^+^ or Tomato^+^ Th1 and Th17 cells were resuspended in restimulation medium in a concentration of 1 × 10^6^ cells/ml and mixed in a 1:1 ratio. The exact ratio between Th1 and Th17 cells was checked by flow cytometry before and after each experiment and the mean between the two measurements was used for quantitative analysis of the videos when correction for the mixing factor was necessary. To prevent fluorescence artifacts on T-cells' behavior, Th1 and Th17 cells were alternatively isolated from 2D2 Ubi-GFP or 2D2 tdTomato in different replicates of the experiment. Accumulation of Th1 and Th17 cells on WT or ICAM-1/-2^−/−^ pMBMECs on the flow chamber was allowed for 5 min at a low shear (0.1 dyn/cm^2^), followed by physiological shear (1.5 dyn/cm^2^) for additional 25 min, for a total recording time of 30 min. Image acquisition was performed at 10 × magnification with an inverted microscope (AxioObserver, Zeiss, Feldbach, Switzerland) with phase contrast and fluorescence illumination using a monochrome charge-coupled device camera (AxioCam MRm Rev, Carl Zeiss). Image analysis was performed using ImageJ software (ImageJ software, National Institute of Health, Bethesda, MD, USA). Numbers of arrested T cells were counted at 20 s after onset of physiological shear. The behavior of arrested T cells was defined and expressed as fractions of categorized T cells set to 100% as follows: T cells that detached during the observation time (detaching); T cells that remained stationary and roundish (stationary); stationary T cells that actively probed the endothelium by sending numerous protrusions (probing); T cells that continuously crawled on the endothelial surface (crawling), T cells that crossed the pMBMECs with prior probing or crawling (probing + diapedesis and crawling + diapedesis); T cells that did not complete a diapedesis events with prior probing or crawling (probing + partial diapedesis and crawling + partial diapedesis). T cells that crawled out of the imaged FOV were not categorized. T-cell crawling tracks were evaluated after manual tracking of individual T cells using the manual tracking plug-in of ImageJ. Distance, speed and forward migration index of crawling tracks were evaluated using chemotaxis and migration tool (version 2.0, Ibidi, Martinsried, Germany).

### Active EAE

Active EAE (aEAE) was induced in 8–12 weeks old female C57BL/6J wild type and ICAM-1/-2^−/−^ C57BL/6J mice exactly as described before (31, 32). Pertussis toxin was applied intraperitoneally (i.p.) either on day 0 (immunization day) and 2 or alternatively on days 1 and 3 post-immunization. Weights and clinical severity were assessed twice daily and scored as (35): 0, asymptomatic; 0.5, limb tail; 1, hind leg weakness; 2, hind leg paraplegia; 3, hind leg paraplegia and incontinence.

### Epifluorescence-IVM of Th1 or Th17 Cell Interaction With the Inflamed Cervical Spinal Cord Microvasculature

Epifluorescence-IVM was performed as described before (34, 36). After visualization of the vascular lumen via injection of 1% tetramethyl rhodamineisothiocyanate (TRITC)-conjugated Dextran into the right carotid artery catheter, 5 × 10^6^ 2D2 GFP Th1 or Th17 cells were infused via the right carotid artery of the anesthetized mouse with a clinical EAE score of 0.5 (limp tail) to 2 (hind leg paraplegia). The shear resistant adhesion of Th1 and Th17 cells to the inflamed spinal cord white matter microvasculature was assessed by scanning multiple field of views (FOVs) of the spinal cord windows at 10, 30, and 60 min after T-cell infusion. The number of permanently adhered Th1 or Th17 cells to the spinal cord microvessels wall of WT or ICAM-1/-2^−/−^ mice was evaluated per each FOV. Off-line data analysis was performed exactly as described before (34).

### 2P-IVM of Th1 and Th17 Cell Interaction With the Inflamed Cervical Spinal Cord Microvasculature

Fluorescent labeling of 2D2 T cells and 2P-IVM imaging of T-cell interactions with the cervical spinal cord microvessels during EAE was done as described previously (37). Briefly, *in vitro* polarized 2D2 CD4^+^ Th1 and Th17 cells were fluorescently labeled with 3 μM CellTracker Green CMFDA (5-chloromethylfluorescein diacetate) or 20 μM CellTracker Blue CMAC (7-amino-4-chloromethylcoumarin) at 37°C for 20 min, with dyes swapped between experiments. After washing, fluorescently labeled Th1 and Th17 cells were mixed 1:1 (5 × 10^6^ from each cell subset) and were systemically injected via the carotid artery catheter into a surgically prepared mouse with a clinical EAE score of 0.5 (limp tail) to 2 (hind leg paraplegia). Fifteen minutes after T-cell transfer, 2P-IVM imaging was performed using the TrimScope system equipped with an Olympus BX50WI fluorescence microscope and a 20 × objective (NA 0.95; LaVision Biotec). 11–15 z-stacks (spacing 4 μm) of 200–400 × 200–400 μm x–y sections were acquired every 20 s for 20–30 min. Blood vessels were labeled with fluorescently labeled (Alexa Fluor 633/Alexa Fluor 594) rat-anti-mouse endoglin antibody (CD105: 2 μg/mouse). As previously showed, endoglin antibody does not interfere with the immune cells interaction with the CNS microvessels (34, 36). The blood vessels with surgical trauma that may have inadvertently occurred during the surgical procedure were excluded from imaging. CellTracker Blue (CMAC) or CellTracker Green (CMFDA) labeled Th1 or Th17 cells, together with Alexa Fluor 633/Alexa Fluor 594-endoglin were excited at 780 nm using a tunable MaiTai HP laser (Spectra Physics). Sequences of image stacks were transformed into volume-rendered four-dimensional image sequences with appropriate software (e.g., Volocity from Perkin Elmer), which was also used for semi-automated tracking of cell motility in three dimensions. For visualization, the image sequences were adjusted for brightness, contrast, and background noise by using appropriate software and the image sequences were exported in QuickTime format.

### Transfer EAE (tEAE)

Th1-cell mediated tEAE was induced as described before (38). Briefly, spleen and pLNs (i.e., axillary, brachial and inguinal LNs) of 2D2 C57BL/6J mice were harvested and CD4^+^ T cells were purified and stimulated *in vitro* with 20 μg/ml of MOG_aa35−55_ peptide (GenScript) in the presence of 20 ng/ml IL-12, 1% IL-2-containing cell supernatant and irradiated syngeneic splenocytes in restimulation medium. On day 6, cells were washed, and viable cells re-stimulated under the same conditions. On day 9, Ficoll-purified Th1 cells (5 × 10^6^/mouse) were injected i.p. into WT and ICAM-1/-2^−/−^ recipient C57BL/6J mice. Weights and clinical severity were assessed twice daily and scored as: 0, asymptomatic; 0.5, limb tail; 1, hind leg weakness; 2, hind leg paraplegia; 3, hind leg paraplegia and incontinence. Additionally, cytokine profile and expression of integrins important for T-cell trafficking was assessed by flow cytometry (Supplementary Figures 6A,B).

Th17-cell mediated tEAE was induced as described before (39, 40). For passive induction of EAE induced via transfer of Th17 cells, naïve 2D2 CD4^+^ T cells were purified using EasySep mouse CD4^+^ T cell isolation kit (STEMCELL™ Technologies). Naïve CD4^+^ T cells were cultured at a concentration 2 × 10^6^/ml in Clone medium [DMEM (Gibco) supplemented with 1 mM sodium pyruvate, 2 mM L-glutamine, 10 U/ml penicillin-streptomycin, 1% (v/v) non-essential amino acids, 1% (v/v) L-Arginine/L- Asparagine solution (Sigma-Aldrich), 1% (v/v) Folic acid solution (Sigma-Aldrich), 1% (v/v) Vitamin solution (Sigma-Aldrich), 0.05 mM β-mercaptoethanol (Grogg Chemie AG), 10% FBS (Biowest)]. Cells were stimulated in the presence of 1 × 10^7^/ml irradiated splenocytes and 2.5 μg/ml anti-CD3 Ab (clone 145-2C11, BioXCell), 20 μg/ml anti-IL-4 Ab (clone 11B11), 20 μg/ml anti-IFNγ (clone XMG1.2), 3 ng/ml hTGF-β1, 30 ng/ml IL-6. Th17 cells were supplemented with 10 ng/ml IL-23 after 48 h. Then 4 days later, Th17 cells with the concentration of 2 × 10^6^/ml were transferred to the plate bound anti-CD3 Ab (2 μg/ml; clone 145-2C11, BioXCell) and anti-CD28 Ab (2 μg/ml; clone PV-1, BioXCell). In parallel, production of IL-17 and RORγt expression was evaluated via flow cytometry. After 2 days, re-stimulated CD4^+^ Th17 cells were collected and extensively washed with PBS. 4 × 10^6^ IL-17-producing cells were injected i.p. into WT and ICAM-1/-2^−/−^ C57BL/6J recipient mice. Weights and clinical severity of mice were assessed twice daily and development of typical and atypical signs (40, 41) of EAE was monitored according to the following criteria: 0: no disease; 1: decreased tail tonus (typical sign) or mild balance defects (atypical sign); 2: hind limb weakness, partial paralysis (typical sign) or severe balance defects that cause spontaneous falling over (atypical sign); 3: complete hind limb paralysis (typical sign) or very severe balance defects that prevent walking (atypical sign); 4: front and hind limb paralysis (typical sign) or inability to move body weight into a different position (atypical sign); and 5: moribund state. Cytokine profile and expression of integrins important for T-cell trafficking was assessed by flow cytometry (Supplementary Figures 6C,D).

### CD45^+^ Inflammatory Cells Isolation From the Brain and Spinal Cord of EAE Mice

Inflammatory cells from the CNS were isolated from each individual mouse at the peak of disease (day 16–20 post-transfer of Th17 cells). Anesthetized mice were perfused with 20 ml of cold PBS (4°C) and brain and spinal cord were dissected from each individual mouse. Tissues were digested with 0.4 mg/ml Collagenase VIII (Sigma-Aldrich) and 2 U/ml DNaseI (Sigma-Aldrich) at 37°C for 30 min. Infiltrated cells were isolated by passing the cell suspension through a 70-μm cell strainer and 33% Percoll density gradient centrifugation. After removal of myelin layer, the infiltrated cells were collected, washed and resuspended in culture medium for further analysis. Cells were resuspended in Fc-block (anti CD16/32 purified from hybridoma supernatants; clone 2.4G2) diluted in PBS for 15 min on ice. Surface staining of T cells was performed with antibodies to CD45 (clone; 30F11, BioLegend), CD4 (clone; RM4-5, BD Pharmingen) in the presence of Fixable Viability Dyes (FVD). After stimulation with PMA/Ionomycin/Golgi stop, the intracellular staining was done using the antibodies to IFN-γ (clone XMG1.2, eBioscience), IL-17 (clone: TC11-18H10, BD Bioscience) and GM-CSF (clone: MP1-22E9, BioLegend). Gating strategy used is shown is Supplementary Figure 6E.

### Statistics

Statistical analysis was performed using GraphPad Prism 6.0 software (Graphpad software, La Jolla, CA, USA). Data were compared by Mann-Whitney test, Student's *t*-test, ANOVA-one way test followed by Bonferroni's post-test or repeated measure ANOVA with Tukey post-test. Data are presented as mean ± SEM and asterisks indicate significant differences (^*^p < 0.05, ^**^p < 0.01 and ^***^p < 0.001, ^****^p < 0.0001).

## Results

### ICAM-1/-2^−/−^ DCs Show Increased Migration Speed and Reduced Interaction Times With Naïve 2D2 GFP T Cells When Compared to WT DCs in Peripheral Lymph Nodes *in vivo*

**Figure 1:**
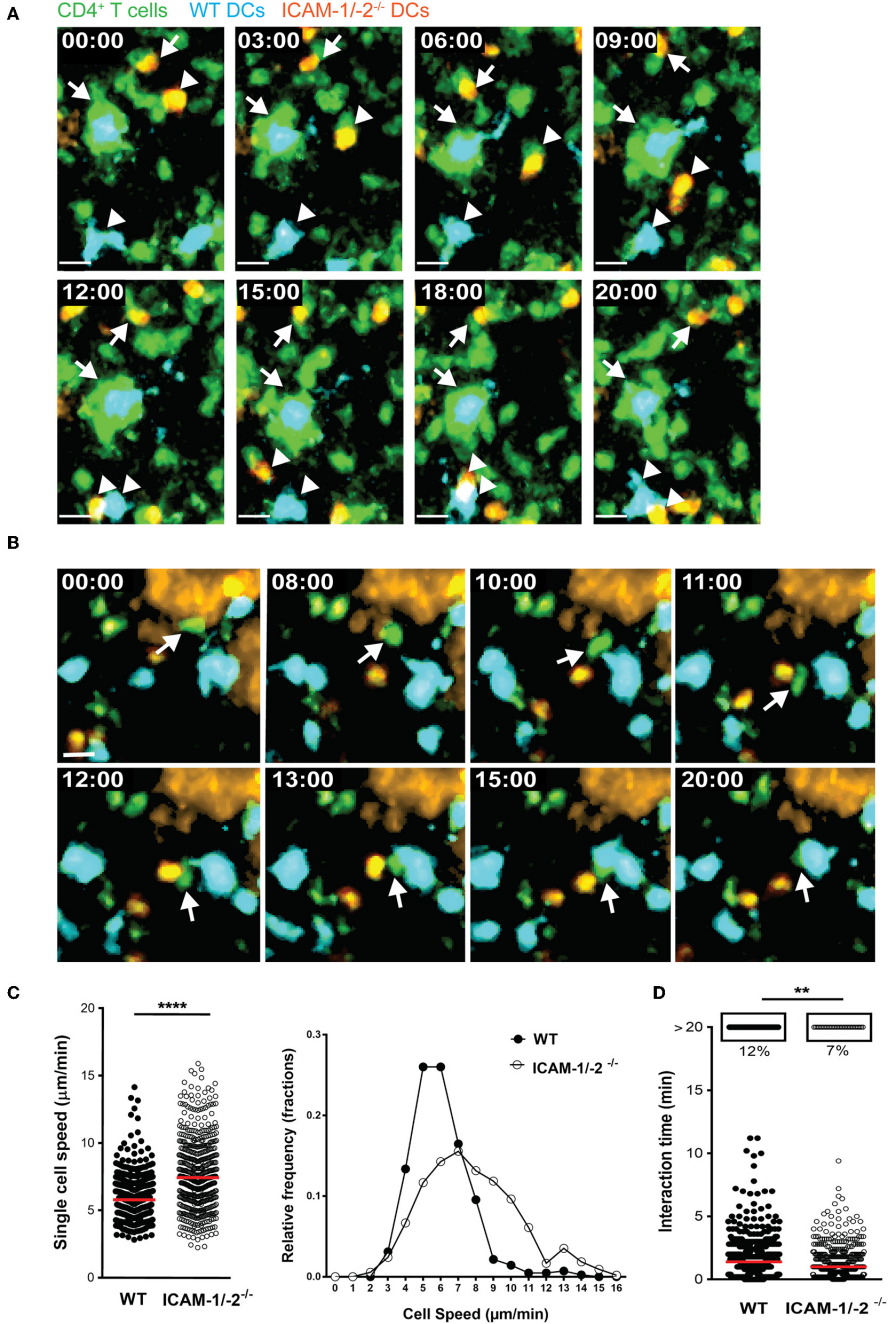
ICAM-1/-2^−/−^ DCs display increased migration speed and shorter interaction times with 2D2 CD4^+^ T cells in popliteal LN *in vivo*. **(A–D)** WT and ICAM-1/-2^−/−^ DCs pulsed with 100 μg/ml MOG_aa35−55_ peptide were subcutaneously injected into WT C57BL/6J mice and allowed to home for 18 h to the dLN. Naïve 2D2-GFP^+^ CD4^+^ T cells were adoptively transferred into recipient mice and their interaction with DCs in the popliteal LN were imaged starting within 10–15 min after injection. **(A,B)** Representative 2P-IVM images showing the interaction of WT and ICAM-1/-2^−/−^ DCs with 2D2-GFP CD4^+^ T cells. **(A)** Arrows indicate a WT DC (blue) and an ICAM-1/-2^−/−^ DC (red) interacting with 2D2-GFP CD4^+^ T cells during the entire time of imaging (20 min). Arrowheads show a transient interaction of a WT DC (blue) and an ICAM-1/-2^−/−^ DC (red) with 2D2-GFP CD4^+^ T cells during the 20 min recording. Time shown in minutes and seconds. Scale bar, 10 μm. **(B)** Arrow points to a 2D2 GFP CD4^+^ T cell positioned between one WT DC (blue) and one ICAM-1/-2^−/−^ DC (red) after migration across the HEV and subsequently interacting preferentially with the WT DC. High endothelial venule, labeled with Alexa Fluor 633–conjugated MECA-79 on the top right corner. Time shown in minutes and seconds. Scale bar, 10 μm. **(C)** Graphs show speed of individual WT and ICAM-1/-2^−/−^ DCs within the PLN and relative frequency of the speeds of the DCs as monitored by 2P-IVM imaging. **(D)** Interaction times between 2D2-GFP CD4^+^ T cells and WT or ICAM-1/-2^−/−^ DCs were monitored. Each dot represents one interaction **(C)** or one track **(D)** quantified over the entire 20 min of recorded movies using Volocity software. Data in **(C)** and **(D)** are pooled from two independent experiments and shown as median. Data were analyzed by unpaired Student's *t*-test. ^**^p < 0.01, ^****^p < 0.0001.

To explore the role of ICAM-1 and ICAM-2 on DCs in activating myelin-specific T cells, we first asked if MOG_aa35−55_ pulsed DCs lacking ICAM-1 and ICAM-2 display visible differences in their dynamic interactions with naïve CD4^+^ T cells compared to WT DCs in peripheral LNs. Naïve CD4^+^ T cells were purified from TCR transgenic 2D2 C57BL/6J mice expressing GFP, in which CD4^+^ T cells recognize MOG_aa35−55_ in the context of I-A^b^. Side-by-side comparison of the dynamic interactions of 2D2 T cells with WT or ICAM-1/-2^−/−^ DCs using two-photon intravital microscopy (2P-IVM) of popliteal LNs (42, 43), showed that 2D2-GFP CD4^+^ T cells preferentially interacted with WT rather than ICAM-1/-2^−/−^ DCs (Figures 1A,B; Supplementary Video 1). Hereby the T cells engaged in longer interactions (≥20 min) with WT DCs than with ICAM-1/-2^−/−^ DCs (Figure 1D and Supplementary Video 2). In accordance to this, MOG_aa35−55_-pulsed WT DCs moved with a significantly lower speed compared to ICAM-1/-2^−/−^ DCs within the LN (Figure 1C; Supplementary Video 2). Thus, absence of ICAM-1 and ICAM-2 reduces the ability of MOG_aa35−55_-pulsed DCs in establishing long-lasting interactions with naïve 2D2 T cells and enhanced their migration speed in the LNs. Thus, ICAMs on DCs mediate the peripheral activation of autoreactive T cells, during initiation of EAE.

### ICAM-1/-2^−/−^ DCs Show Impaired Ability to Fully Activate Naïve 2D2 T Cells *in vivo*

**Figure 2:**
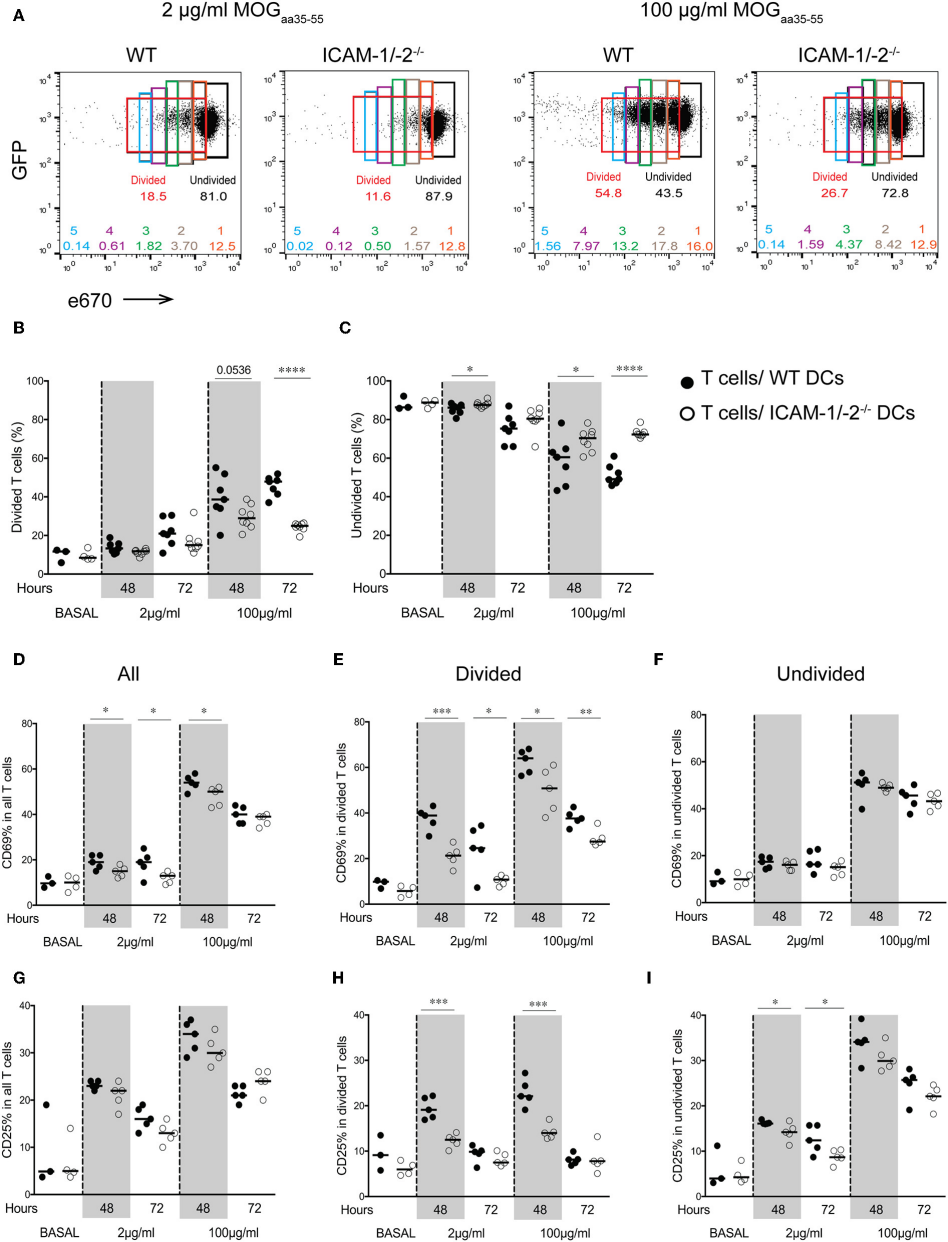
Important role of ICAM-1 and ICAM-2 on DCs in MOG_aa35−55_-specific T-cell proliferation and activation in peripheral lymph nodes of C57BL/6J mice. **(A–I)** Proliferation of e670-labeled 2D2-GFP CD4^+^ T cells 48 and 72 h after transfer into recipient mice prior injected with MOG_aa35−55_-pulsed WT or ICAM-1/-2^−/−^ DCs. DCs were pulsed with 2, 100 μg/ml, or no (BASAL) MOG_aa35−55_ peptide. **(A)** Representative FACS dot plots showing percentage of divided, undivided and each individual generation of divided 2D2-GFP CD4^+^ T cells (generations 1–5) 48 h after adoptive transfer into recipient mice. Gates and the corresponding percentages of undivided cells shown in black, total divided cells in red, generation 1 of divided cells in orange, generation 2 in brown, generation 3 in green, generation 4 in purple, and generation 5 in blue. **(B,C)** Quantification of divided **(B)** or undivided **(C)** e670-labeled 2D2 GFP CD4^+^ T cells percentage after *in vivo* interaction with WT or ICAM-1/-2^−/−^ DCs pulsed with 2, 100 μg/ml, or no (BASAL) MOG_aa35−55_ peptide. Cell divisions were monitored via dilution of the e670 fluorescent dye, 48 and 72 h after transfer of e670-labeled 2D2 GFP CD4^+^ T cells into recipient mice. Each dot represents pLN T cells pooled from one mouse. Data pooled from two individual experiments with a total of 7–8 mice at each time point and 3–4 mice at BASAL level. **(D–I)** Cell surface expression of CD69 and CD25 within all **(D,G)**, divided **(E,H)**, and undivided **(F,I)** 2D2 GFP CD4^+^ T cell-subsets shown as percentage after *in vivo* interaction with WT or ICAM-1/-2^−/−^ DCs pulsed with 2, 100 μg/ml, or no (BASAL) MOG_aa35−55_ peptide. Each dot in the plots represents pLN T cells pooled from one mouse. Results are representative data from two separate experiments with 5 mice in each group (except BASAL level with 3–4 mice) and shown as mean. Data were analyzed using repeated measure ANOVA with Bonferroni post-test. ^*^p < 0.05, ^**^p < 0.01, ^***^p < 0.001, ^****^p < 0.0001.

We next asked how lack of ICAMs on DCs will influence CD4^+^ 2D2 T cell activation *in vivo*. To this end, we pulsed LPS-matured ICAM-1/-2^−/−^ or WT DCs with low (2 μg/ml) or high (100 μg/ml) concentrations of MOG_aa35−55_ peptide or no peptide, and allowed them after subcutaneous (s.c.) injection into WT C57BL/6J mice to reach the dLNs. Eighteen hours after DCs injection, naïve e670-labeled 2D2-GFP CD4^+^ T cells were injected and allowed to home to the dLNs and interact with WT and ICAM-1/2^−/−^ DCs. 2D2 T-cell activation and proliferation was determined at 48 and 72 h after their injection. MOG_aa35−55_-induced division of 2D2 T cells *in vivo* was found to be significantly reduced after encounter with ICAM-1/-2^−/−^ DCs compared to WT DCs at 48 and 72 h (Figures 2A,B). Interestingly, reduced T-cell proliferation induced by ICAM-1/-2^−/−^ DCs was already observed from the second generation of proliferated CD4^+^ T cells (Supplementary Figure 1B). Accordingly, the percentage of undivided 2D2 T cells isolated from the LNs of mice having received MOG_aa35−55_ pulsed ICAM-1/-2^−/−^ DCs was significantly higher compared to mice that had received MOG_aa35−55_ pulsed WT DCs (Figure 2C). Reduced 2D2 T-cells proliferation induced by ICAM-1/-2^−/−^ vs. WT DCs correlated to their reduced activation highlighted by reduced cell surface expression of the early activation markers CD69 (Figures 2D,E; Supplementary Figure 1C) and CD25 (Figures 2G,H; Supplementary Figure 1C) on the proliferating 2D2 T cells. Undivided 2D2 T cells did not shown any major differences in CD25 and CD69 expression (Figures 2F,I). Thus, ICAM-1/-2^−/−^ DCs promote reduced *in vivo* proliferation of 2D2 CD4^+^ T cells due to their failure in inducing full T-cell activation.

### ICAM-1 but Not ICAM-2 Is Required on Antigen Presenting Cells for Antigen-Specific Naïve CD4^+^ T-Cell Activation

**Figure 3:**
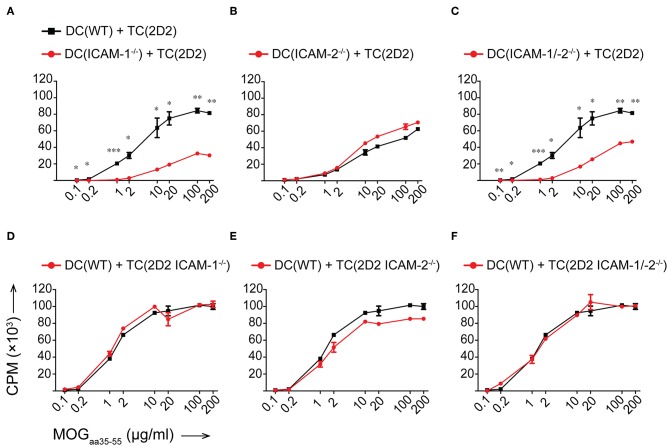
Reduced *in vitro* MOG_aa35−55_-specific CD4^+^ T-cell proliferation in the absence of ICAM-1 but not ICAM-2 on DCs. **(A–F)** Purified CD4^+^ T cells (TC) were co-cultured with irradiated BM-derived DCs (DC) with a ratio of 1:10 DCs:TCs. Different concentrations of MOG_aa35−55_ peptide were added to the co-cultures and incubated for 72 h before pulsing with [^3^H]-thymidine. ^3^H-thymidine incorporation into TCs shown as counts per minute (CPM)/well. **(A–C)** TCs from 2D2 C57BL/6J mice co-cultured with irradiated DCs isolated from WT, ICAM-1^−/−^ **(A)**, ICAM-2^−/−^ **(B)**, or ICAM-1/-2^−/−^ **(C)** C57BL/6J mice. **(D–F)** TCs from 2D2, 2D2 ICAM-1^−/−^ **(D)**, 2D2 ICAM-2^−/−^ **(E)** or 2D2 ICAM-1/-2^−/−^ **(F)** C57BL/6J mice co-cultured with irradiated DCs from WT C57BL/6J mice. Results show mean ± SEM after subtraction of background proliferation determined in the absence of MOG_aa35−55_ peptide. Data are representative of 3-4 individual experiments per condition. Statistical differences between the groups calculated with paired Student's *t*-test. ^*^p < 0.05, ^**^p < 0.01, ^***^p < 0.001.

To determine if ICAM-1 and ICAM-2 play redundant roles on DCs in activating myelin-specific CD4^+^ T cells, we next differentiated DCs from the bone marrow of WT, ICAM-1^−/−^, ICAM-2^−/−^, and ICAM-1/-2^−/−^ C57BL/6J mice and ensured that LPS maturation induced comparable maturation and activation (Supplementary Figure 2A). Co-incubation of 2D2 T cells with matured WT DCs in the presence of increasing concentrations of MOG_aa35−55_ peptide induced a dose dependent proliferation of naïve 2D2 CD4^+^ T cells (Figure 3A). While ICAM-1^−/−^ DCs failed to induce efficient 2D2 T-cell proliferation, ICAM-2^−/−^ DCs induced MOG-specific 2D2 T-cell proliferation comparable to WT DCs (Figures 3A,B). Lack of both ICAMs on DCs did not lead to reduced MOG-specific 2D2 T cell proliferation, when compared to ICAM-1^−/−^ DCs (Figure 3C). We next explored the contribution of ICAM-1 and ICAM-2 on a mixed population of APCs, including macrophages and B cells, by employing lethally irradiated splenocytes harvested from WT, ICAM-1^−/−^, ICAM-2^−/−^, or ICAM-1/-2^−/−^ C57BL/6J mice. We confirmed lack of involvement of ICAM-2 on mixed APCs on the MOG_aa35−55_ dose dependent proliferation of 2D2 T cells (Supplementary Figures 2B–D). Involvement of T-cell expressed ICAM-1 and ICAM-2 in Ag-specific activation of naïve 2D2 CD4^+^ T cells was excluded as co-incubation of WT DCs (Figures 3D–F) or WT APCs (Supplementary Figures 2E–G) with ICAM-1^−/−^, ICAM-2^−/−^, and ICAM-1/-2^−/−^ 2D2 T cells did not impair their MOG_aa35−55_-peptide dose dependent proliferation or induction of signature cytokines when compared to WT 2D2 T cells (Supplementary Figures 3A,B).

Taken together, ICAM-1 and ICAM-2 on T cells are not required for the Ag-specific activation of naïve CD4^+^ T cells. Rather, on APCs, ICAM-1 but not ICAM-2 contributes to the IS formation required for the sufficient duration of T cell/DC interactions leading to full Ag-specific T-cell activation and proliferation of myelin-specific CD4^+^ T cells. Thus, ICAM-1 but not ICAM-2 plays an important role in early EAE pathogenesis during the priming of autoaggressive CD4^+^ T cells.

### Brain Endothelial ICAM-1 and ICAM-2 Are Essential for Sustained Crawling of Th1 and Th17 Cells on the BBB *in vitro*

Identifying non-redundant roles for ICAM-1 and ICAM-2 in the initiation of EAE pathogenesis we next addressed the role of ICAM-1 and ICAM-2 in the effector phase of the disease, where encephalitogenic CD4^+^ T cells cross the BBB and upon reaching the CNS parenchyma trigger the clinical disease. To clarify the role of endothelial ICAM-1 and ICAM-2 in mediating Th1 vs. Th17 cells migration across the BBB, we first compared side-by-side the multistep extravasation of myelin-specific Th1 and Th17 cells across WT or ICAM-1/-2^−/−^ primary mouse brain microvascular endothelial cells (pMBMECs) under physiological flow by *in vitro* live cell imaging. We stimulated pMBMECs with TNFα or IL-1β to induce low and high expression of ICAM-1 and vascular cell adhesion molecule (VCAM)-1, respectively, while ICAM-2 is constitutively expressed under both conditions (14).

**Figure 4:**
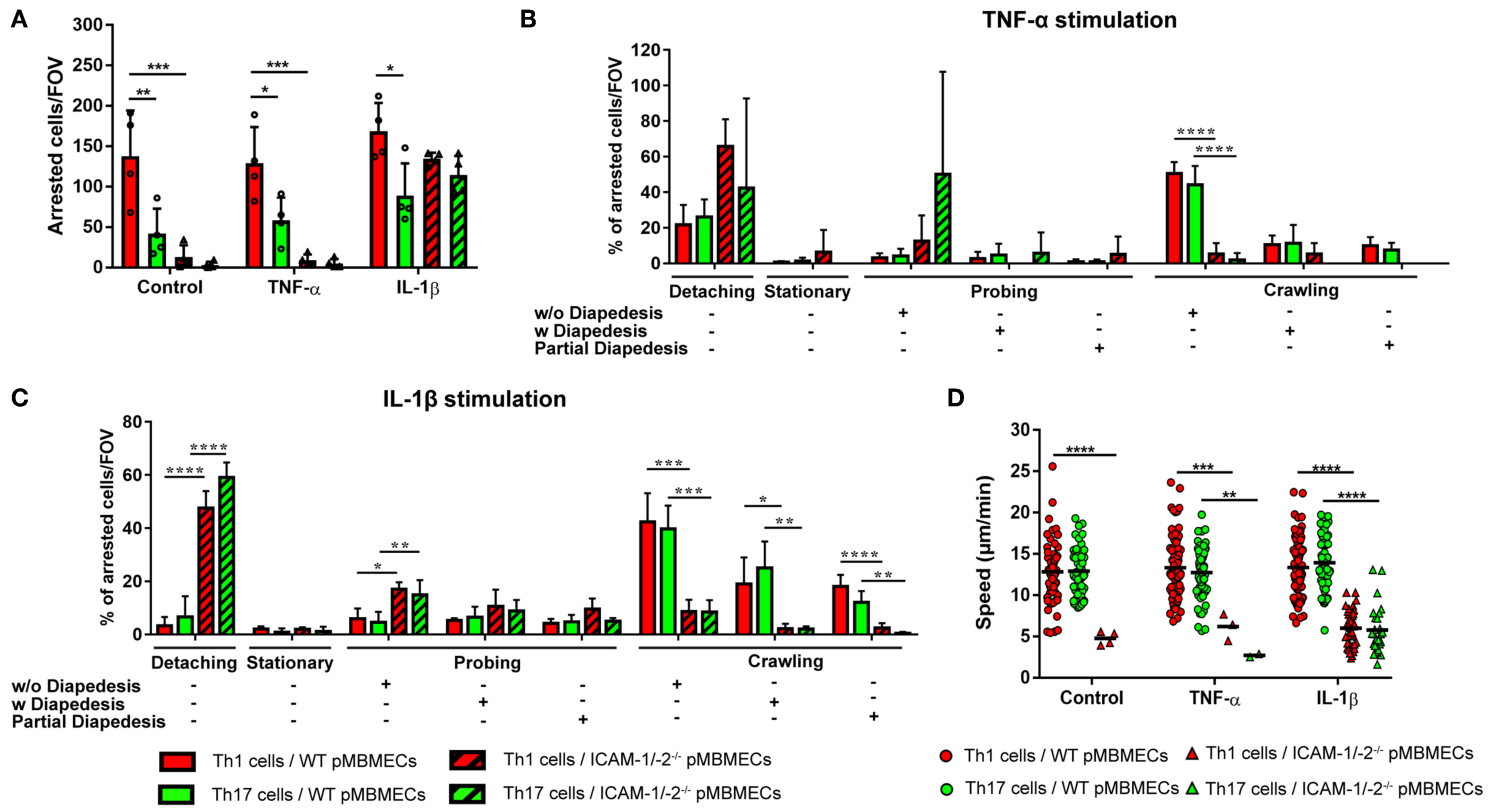
Absence of ICAM-1 and ICAM-2 affects the interaction of *in vitro* polarized Th1 and Th17 CD4^+^ cells with an *in vitro* model of the BBB. **(A)** Mean number of arrested CD4^+^ Th1 (red) and Th17 (green) cells per FOV (872 × 654 μm) on WT (filled bars) and ICAM-1/-2^−/−^ (striped bars) non-stimulated (control), TNFα or IL-1β-stimulated pMBMECs. Each dot represents the number of arrested T cells in one experiment, for a total of four independent experiments. Data shown as mean ± SD. **(B,C)** Post-arrest dynamic behavior during 30 min of recording time of *in vitro* polarized CD4^+^ Th1 and Th17 cells on TNFα **(B)** or IL-1β **(C)** stimulated pMBMECs. Presence or absence of complete or partial diapedesis is indicated for each migratory behavioral category, as indicated. The behavioral categories are presented as percentage of categorized CD4^+^ Th1 and Th17 cells for each condition of pMBMECs. Data shown as mean ± SD from four independent experiments. **(D)** Crawling speed in μm/min of CD4^+^ Th1 and Th17 cells on WT (squares) and ICAM-1/-2^−/−^ (triangles) control, TNFα or IL-1β-stimulated pMBMECs. Each point represents one crawling track of one CD4^+^ Th1 or Th17 cell. Values shown as mean (Th1 or Th17 crawling tracks on WT pMBMECs ≥50; Th1 or Th17 crawling tracks on ICAM-1/-2^−/−^ pMBMECs ≤ 37) and are pooled from four individual experiments. Data were analyzed using repeated measure ANOVA with Tukey post-test ^*^p < 0.05, ^**^p < 0.01, ^***^p < 0.001, ^****^p < 0.0001.

Irrespective of the inflammatory status, the number of Th1 cells arresting on WT pMBMECs under physiological flow was significantly higher compared to Th17 cells (Figure 4A), suggesting intrinsic superior adhesive characteristics of Th1 over Th17 cells. Analysis of the post-arrest behavior of Th1 and Th17 cells interacting with WT pMBMECs revealed that the inflammatory phenotype of the pMBMECs rather than the identity of the Th subset influenced the multi-step interaction cascade. Increasing inflammatory conditions of pMBMECs (no stimulation to TNFα to IL-1β stimulation) equally strengthened the interaction of both Th cell subsets with pMBMECs, as shown by reduced T-cell detachment and concomitant increase in T-cell crawling and diapedesis (Supplementary Figure 5A, Figures 4B,C, Supplementary Video 3).

Absence of endothelial ICAM-1 and ICAM-2 dramatically reduced the arrest of both Th1 and Th17 cells on pMBMECs, without observed differences between the two Th cell subsets. Under conditions of no or low inflammation (no or TNFα stimulation) lack of endothelial ICAM-1 and ICAM-2 entirely abrogated the arrest of Th1 and Th17 cells on pMBMECs. However, under conditions of high inflammation (Il-1β stimulation), when endothelial VCAM-1 is highly expressed (14), ICAM-1/-2^−/−^ pMBMECs could support Th1 and Th17 cells arrest. Lack of endothelial ICAM-1 and ICAM-2 significantly altered Th1 and Th17 cells post-arrest behavior, again in a comparable manner. The vast majority of Th1 and Th17 cells able to arrest on IL-1β stimulated ICAM-1/-2^−/−^ pMBMECs were unable to polarize and resist shear forces (Supplementary Video 4), resulting in their detachment (Figure 4C). Th1 and Th17 cells that did not detach from ICAM-1/-2^−/−^ pMBMECs showed impaired crawling on pMBMECs (Supplementary Figure 5A, Figures 4B,C, Supplementary Video 4), as also reflected by their reduced crawling speed (Figure 4D) and crawling distance (Supplementary Figure 5B). In addition, IL-1β stimulated ICAM-1/-2^−/−^ pMBMECs increased Th1 and Th17-cell crawling directionality along the direction of flow, while Th1 and Th17-cell crawling on WT pMBMECs was observed also against the flow (Supplementary Figure 5C). Finally, diapedesis of both Th1 and Th17 cells across IL-1β-stimulated ICAM-1/-2^−/−^ pMBMECs was significantly reduced when compared to WT pMBMECs (Figure 4C). Interestingly, we observed a significant increase in the probing behavior of both Th1 and Th17 cells on IL-1β-stimulated ICAM-1/-2^−/−^ vs. WT pMBMECs, suggesting that reduced T-cell crawling in the absence of ICAM-1 and ICAM-2 may favor T-cell probing on brain endothelium (Figure 4C). Taken together, with the exception to the increased ability of Th1 vs. Th17 cells to arrest on pMBMECs under flow *in vitro*, we did not observe any cell type specific behavior of Th1 vs. Th17 cells with the *in vitro* BBB model and endothelial ICAM-1 and ICAM-2 were found equally important for the migration of Th1 and Th17 cells across the BBB.

### Endothelial ICAM-1 and ICAM-2 Mediate Crawling of Th1 and Th17 Cells on the Inflamed BBB *in vivo*

**Figure 5:**
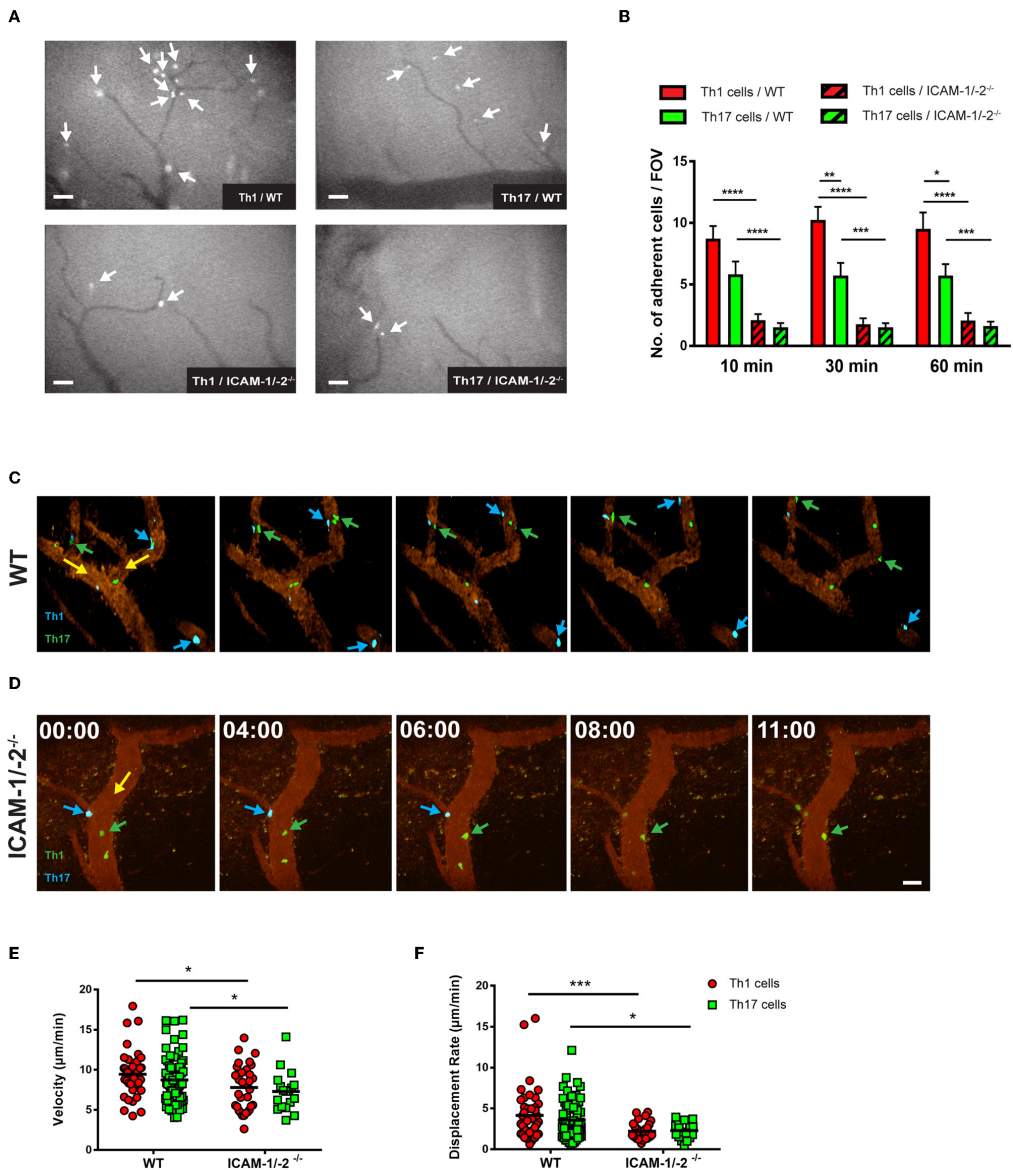
Lack of ICAM-1 and ICAM-2 impairs firm adhesion and crawling of *in vitro* polarized Th1 and Th17 CD4^+^ cells with the inflamed BBB *in vivo*. **(A)** Representative frames of epifluorescence-IVM imaging of Th1 or Th17 cells adhering in inflamed cervical spinal cord microvessels of WT or ICAM-1/-2^−/−^ C57BL/6J mice with EAE 60 min post-infusion. White arrows indicate arrested Th1 or Th17 cells in cervical spinal cord microvasculature. **(B)** Number of adhered Th1 and Th17 cells in inflamed cervical spinal cord microvessels of WT and ICAM-1/-2^−/−^ C57BL/6J mice with EAE counted at 10, 30, and 60 min after infusion of Th1 and Th17 cells. Data are pooled from imaging of 4 EAE mice per each condition and are shown as mean ± SEM. **(C,D)** Movement of fluorescently labeled Th1 and Th17 CD4^+^ cells on the inflamed BBB of WT **(C)** or ICAM-1/-2^−/−^ **(D)** EAE mice is shown at 0, 4, 6, 8, and 11 min of recording. Vascular endothelial cells were labeled by infusion of fluorophore-conjugated rat-anti-mouse endoglin antibody (CD105: 2 μg/mouse). Time shown in minutes and seconds. Scale bar: 40 μm. **(C)** Blue arrows indicate CellTracker Blue (CMAC) labeled Th1 cells and green arrows show CellTracker Green (CMFDA) labeled Th17 cells interacting with spinal cord microvessels of WT C57BL/6J mice with EAE. Both Th1 and Th17 cells crawled with and against the direction of blood flow (yellow arrow). **(D)** Green arrow shows CMFDA labeled Th1 cells; blue arrow shows CMAC labeled Th17 cells interacting with spinal cord microvessels of ICAM-1/-2^−/−^ C57BL/6J mice during EAE. Adhering Th1 and Th17 cells on the inflamed BBB either detached (yellow arrow) and recirculated or failed to crawl in inflamed ICAM-1/-2^−/−^ microvessels during EAE. **(E,F)** Intraluminal crawling of fluorescently labeled Th1 or Th17 cells on endothelial cells of inflamed spinal cord microvessels were imaged using time-lapse 2P-IVM. Each data point represents one individual CD4^+^ Th1 or Th17 cells track. Speed **(E)** and displacement rate **(F)** of individual interacting Th1 and Th17 CD4^+^ cells with inflamed BBB of WT and ICAM-1/-2^−/−^ C57BL/6J mice suffering from EAE were calculated using Volocity software. Values are pooled from imaging of 4 EAE mice per each condition and are shown as ± SEM. Data were analyzed using Mann-Whitney-Test to compare each two groups ^*^p < 0.05, ^**^p < 0.01, ^***^p < 0.001, ^****^p < 0.0001.

We next asked if lack of endothelial ICAM-1 and ICAM-2 will equally affect the interaction of myelin-specific Th1 and Th17 cells with the BBB *in vivo*. Employing real-time epifluorescence intravital microscopy (IVM) to observe the initial interaction of Th1 or Th17 cells with the vascular wall in spinal cord microvessels in WT and ICAM-1/-2^−/−^ C57BL/6J mice suffering from EAE we did not observe any difference in Th1 and Th17 cells initial interactions (capture + rolling) in the absence or presence of ICAM-1 and ICAM-2 (data not shown). In accordance to our observations *in vitro*, significantly higher numbers of Th1 cells compared to Th17 cells were observed to arrest within the inflamed spinal cord microvessels of WT mice (Figures 5A,B) and absence of ICAM-1 and ICAM-2 almost completely abrogated arrest of both Th1 and Th17 cells (Figures 5A,B). Thus, endothelial ICAM-1 and ICAM-2 mediate shear resistant adhesion of both Th1 and Th17 cells to the inflamed BBB *in vivo*.

To explore the role of endothelial ICAM-1 and ICAM-2 in post-arrest Th1 and Th17-cell interaction with the inflamed BBB *in vivo*, we next took advantage of 2P-IVM of spinal cord microvessels in WT and ICAM-1/-2^−/−^ C57BL/6J mice with EAE. Th1 and Th17 cells showed equal ability to crawl along and against the direction of blood flow in spinal cord microvessels in WT mice with EAE (Figure 5C, Supplementary Video 5). In contrast, lack of ICAM-1 and ICAM-2 significantly reduced crawling of Th1 and Th17 cells that still were able to arrest within ICAM-1/-2^−/−^ spinal cord microvessels (Figure 5D, Supplementary Video 6). This was accompanied by a significant reduction in their crawling speed and displacement rate when compared to Th1 and Th17-cell crawling within WT spinal cord microvessels (Figures 5E,F). Our *in vitro* and *in vivo* observations underscore that besides the increased ability of Th1 over Th17 cells to arrest to the BBB, post-arrest behavior of Th1 and Th17 cells on the BBB is comparable and equally affected by the absence of ICAM-1 and ICAM-2 on the BBB.

### Absence of ICAM-1 and ICAM-2 Ameliorates Th1 and Th17-cell Mediated Typical and Atypical EAE

**Figure 6:**
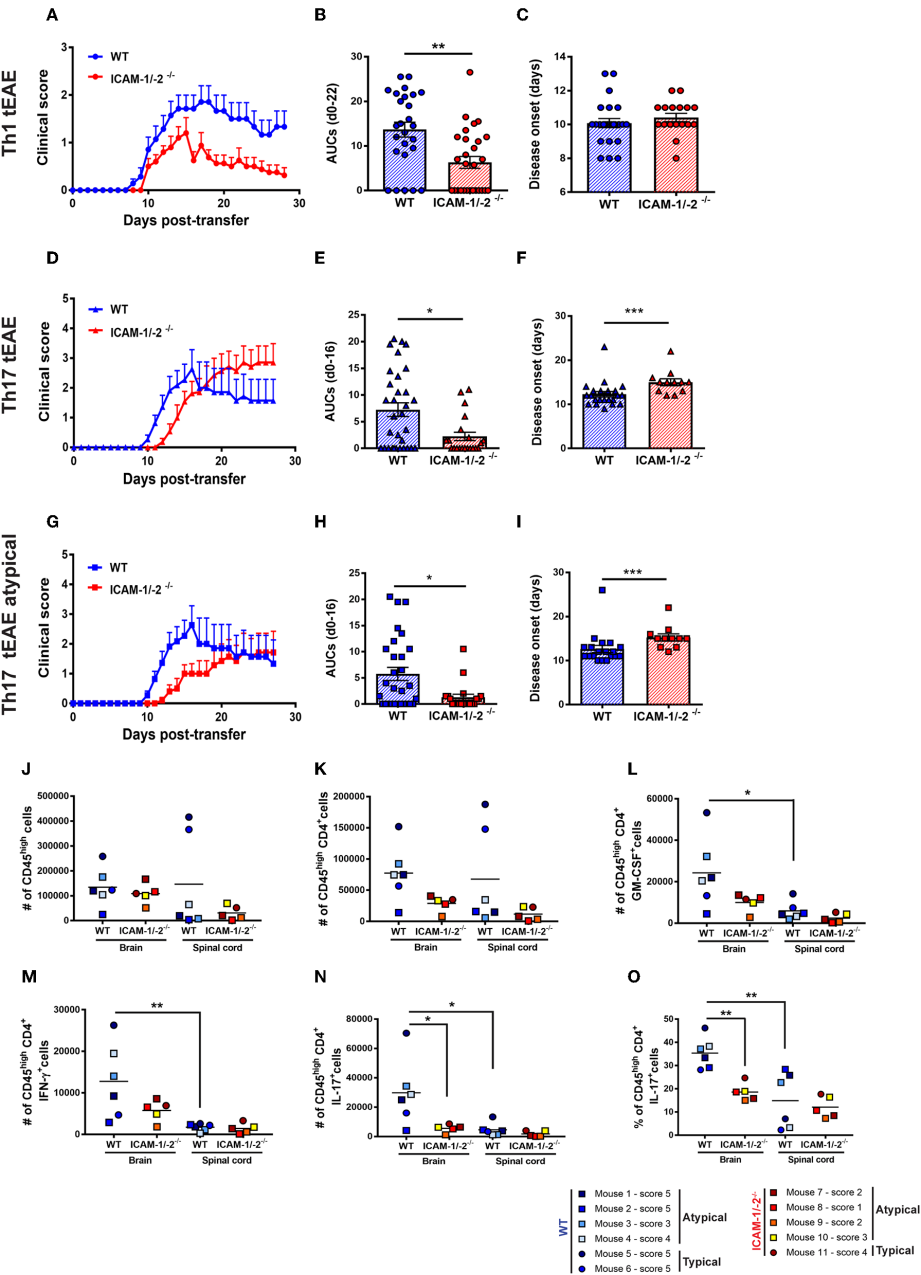
Absence of ICAM-1 and ICAM-2 ameliorates Th1 and Th17 mediated EAE and reduces brain infiltration of cytokine-producing T cells. **(A–C)** Representative clinical course **(A)**, sum of the area under the curve (AUC) **(B)**, and disease onset **(C)** of Th1 cell mediated typical EAE (circles) in WT (blue) and ICAM-1/-2^−/−^ (red) mice. **(A)** Representative clinical course of Th1 cell mediated EAE in WT and ICAM-1/-2^−/−^ mice. Clinical disease course is shown as mean ± SEM of 7 WT mice and 10 ICAM-1/-2^−/−^ mice. **(B)** Severity of disease was compared by the sum of the AUC of typical Th1 tEAE until day 22 post-transfer, all animals stayed alive during the entire time period. Results are shown as mean ± SEM and are pooled from 4 to 6 experiments comparing a total of 26 WT mice and 28 ICAM-1/-2^−/−^ mice. Each dot represents one mouse. **(C)** Mean day of disease onset of Th1 tEAE between WT and ICAM-1/-2^−/−^ recipient mice shown as mean ± SEM. Data are pooled from 4 to 6 experiments (29 WT mice and 30 in ICAM-1/-2^−/−^ mice). Each dot represents one mouse. **(D–F)** Representative clinical course **(D)**, sum of the AUC **(E)**, and disease onset **(F)** of Th17 cell mediated typical and atypical EAE (triangles) in WT (blue) and ICAM-1/-2^−/−^ (red) mice. **(D)** Representative clinical course of Th17 cell mediated typical and atypical EAE in WT and ICAM-1/-2^−/−^ mice. Clinical disease course is shown as mean ± SEM of 11 WT mice and 11 ICAM-1/-2^−/−^ mice. Clinical course is calculated by pooling mice that showed typical and/or atypical (triangles) signs of EAE. **(E)** Severity of disease was compared by the AUC of typical and atypical Th17 tEAE until day 16 post-transfer, all animals stayed alive during the entire time period. Results are shown as mean ± SEM and are pooled from three experiments comparing a total of 27 WT mice and 21 ICAM-1/-2^−/−^ mice. Each dot represents one mouse. **(F)** Mean day of disease onset of typical and atypical Th17 tEAE between WT and ICAM-1/-2^−/−^ recipient mice shown as mean ± SEM. Data are pooled from 2 to 3 experiments (28 WT mice and 23 ICAM-1/-2^−/−^ mice). Each dot represents one mouse. **(G–I)** Representative clinical course **(G)**, sum of the AUC **(H)**, and disease onset **(I)** of Th17 cell mediated atypical EAE (squares) in WT (blue) and ICAM-1/-2^−/−^ (red) mice. **(G)** Representative clinical course of Th17 cell mediated atypical EAE in WT and ICAM-1/-2^−/−^ mice. Clinical disease course is shown as mean ± SEM of 11 WT and 10 ICAM-1/-2^−/−^ mice. Clinical course is calculated on mice that showed atypical signs of EAE. **(H)** Severity of disease was compared by the AUC of atypical Th17 tEAE until day 16 post-transfer, all animals stayed alive during the entire time period. Results are shown as mean ± SEM and are pooled from three experiments comparing a total of 25 WT mice and 20 ICAM-1/-2^−/−^ mice. Each dot represents one mouse. **(I)** Mean day of disease onset of atypical Th17 tEAE between WT and ICAM-1/-2^−/−^ recipient mice shown as mean ± SEM. Data are pooled from 2 to 3 experiments (26 WT mice and 22 in ICAM-1/-2^−/−^ mice). Each dot represents one mouse. **(A–I)** AUC, mean day of disease onset and mean of disease incidence were calculated with Mann-Whitney-Test ^*^p < 0.05, ^**^p < 0.01, ^***^p < 0.001. **(J–N)** Quantification of number of CD45^high^ **(J)**, CD45^high^CD4^+^ **(K)**, CD45^high^CD4^+^GM-CSF^+^ **(L)**, CD45^high^CD4^+^IFN-γ^+^ **(M)**, CD45^high^CD4^+^IL-17^+^ **(N)**, and percentage of CD45^high^CD4^+^IL-17^+^ **(O)** cells evaluated by flow cytometry analysis in brain and spinal cord of WT (blue shades) and ICAM-1/-2^−/−^ (red shades) recipient mice suffering from typical (circles) or atypical (squares) EAE. Each dot represents one individual EAE mouse and a total of 6 WT and 5 ICAM-1/-2^−/−^ mice were analyzed per each group at the peak of disease (day 16–20 post-transfer of *in vitro* polarized CD4^+^ Th17 cells). Data are pooled from two individual experiments and shown as mean. Data analyzed using repeated measure two-way ANOVA with Tukey post-test ^*^p < 0.05, ^**^p < 0.01.

Lack of observing any differences in the involvement of ICAM-1 and ICAM-2 in the migration of Th1 and Th17 cells across the BBB *in vitro* and *in vivo*, we finally asked if absence of ICAM-1 and ICAM-2 also equally affects Th1 and Th17 cells mediated typical and atypical EAE. Adoptive transfer of *in vitro-*polarized encephalitogenic Th1 into ICAM-1/-2^−/−^ C57BL/6J mice recipient mice resulted in a significant amelioration of the disease course when compared to WT C57BL/6J littermates underscored by the reduced area under the curve (AUC) (Figures 6A,B), without affecting disease onset or incidence (Figure 6C and data not shown).

In order to compare if Th17 cells—in contrast to Th1 cells—use different mechanisms to infiltrate in the CNS, we next induced Th17 tEAE by adoptive transfer of *in vitro*-polarized encephalitogenic Th17 cells into C57BL/6J mice. We observed that the overall severity of Th17 cell-induced EAE, as determined by the AUC, was significantly reduced in the ICAM-1/-2^−/−^ recipients compared with WT C57BL/6J mice (Figures 6D,E). Moreover, disease onset was significantly delayed in ICAM-1/-2^−/−^ vs. WT C57BL/6J mice (Figure 6F). We observed no significant difference in disease incidence of ICAM-1/-2^−/−^ and WT C57BL/6J recipients (data not shown). Since atypical signs of EAE have been assigned to Th17-cell infiltration into the brain rather than into the spinal cord (17), we separately evaluated development of atypical EAE. While we did not observe a difference in disease incidence (data not shown), ICAM-1/-2^−/−^ C57BL/6J mice showed delayed onset (Figure 6I) and significant amelioration of atypical EAE (Figures 6G,H) compared to WT recipients. The observed amelioration of Th1 and Th17-cell mediated EAE in ICAM-1/-2^−/−^ mice highlight the important role of ICAM-1 and ICAM-2 in regulating the effector phase of Th1 and Th17-cell mediated EAE.

### Absence of ICAM-1 and ICAM-2 in C57BL/6J Mice Reduces the Homing of CD4^+^ Th17 Cells Into the CNS During EAE

Transfer of encephalitogenic Th17 cells into naïve syngeneic recipients resulted in atypical and typical signs of EAE clinical manifestations that have been attributed to preferential infiltration of T cells into the brain and spinal cord parenchyma, respectively (17, 44). To determine if lack of ICAM-1 and ICAM-2 differentially affects migration of T cells into the brain or spinal cord, we isolated CD45^+^ inflammatory cells from the brain and spinal cord of WT or ICAM-1/-2^−/−^ mice at the peak of typical and atypical Th17-cell mediated tEAE. Irrespective of the EAE phenotypic manifestation, comparable numbers of CD45^high^ infiltrating cells were found in the brain of WT and ICAM-1/-2^−/−^ mice (Figure 6J). In the spinal cord, a trend toward higher numbers of CD45^high^ cells was found in WT mice with typical EAE as compared to atypical EAE (Figure 6J), in accordance with previous findings (45). Absence of ICAM-1 and ICAM-2 slightly reduced infiltration of CD45^high^ cells into the spinal cord in typical EAE, but not in atypical EAE (Figure 6J). Quantification of CNS infiltrating CD4^+^ T cells (Figure 6K) showed a trend toward reduction of the numbers of CD4^+^ T cells in the brain of ICAM-1/-2^−/−^ mice suffering from either typical and atypical EAE. In the spinal cord lack of ICAM-1 and ICAM-2 moderately reduced the number of CD4^+^ T cells in mice with typical but not with atypical EAE (Figure 6K). Despite the low numbers of individual mice investigated, mice suffering from atypical EAE showed higher numbers of CD45^high^ CD4^+^T cells in the brain rather than in the spinal cord, further supporting the notion that brain infiltrating CD4^+^ T cells cause atypical EAE (Figures 6J,K). To further characterize the CNS infiltrating CD4^+^ T cells, we measured in a non-exclusive manner the expression of Th1 and Th17 signature cytokines IFN-γ and IL-17, respectively, in addition to GM-CSF, which has been proposed as a pathogenic mediator in MS and EAE (46–49). The number of cytokine-producing CD4^+^ T cells was significantly higher in the brain when compared to the spinal cord in WT mice in both typical and atypical EAE (Figures 6L–N). This was accompanied by a significantly reduced percentage of CD4^+^ IL-17^+^ spinal cord vs. brain infiltrating T cells (Figure 6O), underscoring that the observed preferential infiltration of CD4^+^ IL-17^+^ cells into the brain is not due to a larger size of the brain vs. the spinal cord. At the same time the percentages of brain and spinal cord infiltrating GM-CSF^+^ and IFN-γ^+^ CD4^+^ T cells were found to be comparable (Supplementary Figures 7A,B). Absence of ICAM-1 and ICAM-2 resulted in a trend toward reduction of the numbers of GM-CSF^+^ and IFN-γ^+^ CD4^+^ brain infiltrating T cells in both typical and atypical EAE (Figures 6L,M). Remarkably, we found significantly lower numbers and percentage of CD4^+^ IL-17^+^ cells in the brain of ICAM-1/-2^−/−^ vs. WT C57BL/6J mice irrespective of typical or atypical signs of (Figures 6N,O), underscoring that absence of ICAM-1 and ICAM-2 preferentially affects brain infiltration by Th17 cells. In addition, in WT mice the number of brain infiltrating IL-17^+^ T cells was 2-fold higher than that of brain infiltrating IFN-γ^+^ T cells in WT mice, underscoring that Th17 cells preferentially infiltrate the brain. As expression of GM-CSF in CD4^+^ Th17 cells has been shown to be essential for their encephalitogenic potential (46), we also analyzed expression of GM-CSF in combination with IL-17 in CD4^+^ T cells infiltrating the brains and spinal cords of WT and ICAM-1/-2^−/−^ mice during EAE. Interestingly, we found both GM-CSF^+^ IL-17^+^ T cells as well as GM-CSF^+^ IL-17^−^ T cells in the CNS of WT mice during EAE (Supplementary Figure 7C). Absence of ICAM-1 and ICAM-2 significantly reduced the numbers of brain infiltrating GM-CSF^+^ IL-17^+^ T cells (Supplementary Figure 7C), which correlates with the reduced clinical severity of EAE in ICAM-1/-2^−/−^ vs. WT mice. Taken together, our present study shows that ICAM-1 but not ICAM-2 on APCs mediates peripheral activation of encephalitogenic T cells during initiation of EAE. In the effector phase, endothelial ICAM-1 and ICAM-2 are essential for Th1 and Th17-cell crawling on the BBB. Absence of ICAM-1 and ICAM-2 resulted in significantly reduced severity of both, Th1 and Th17-cell mediated EAE underscoring the important role for ICAM-1 and ICAM-2 in mediating T-cell trafficking into the brain and spinal cord.

## Discussion

The contribution of ICAM-1 on APCs at the IS in naïve T-cell activation is well-known (8, 50–53), although a role of ICAM-2 on APCs as alternative ligand of LFA-1 has not been fully explored. Here we found that ICAM-1, but not ICAM-2, on DCs and in a mixed population of APCs, is involved in the activation of naïve autoaggressive T cells. In this context it is important to note that the affinity of LFA-1 binding to ICAM-1 is 5-fold higher compared to ICAM-2 (54, 55). It is therefore possible that ICAM-2 alone does not allow for sufficiently long T cell/APC interactions to induce proliferation due to its weaker binding capacity with LFA-1. Compensatory upregulation of other reported LFA-1 ligands, such as ICAM-3, ICAM-4, and ICAM-5 was not investigated as these molecules are not expressed in mouse myeloid cells and endothelial cells (56, 57).

Furthermore, we found no role for T-cell expressed ICAM-1 and ICAM-2 in triggering MOG_aa35−55_ induced T-cell activation, suggesting a precise and non-interchangeable relationship in the molecular interactions between APCs and T cells at the IS. Our observations are in apparent contrast with previous studies failing to identify a role for ICAM-1 and ICAM-2 on DCs in inducing T-cell proliferation (58, 59). Discrepancies between these studies might be due to subtle differences in experimental setups, including use of different DC polarization protocols and the use of different T-cell subsets, which may result in different TCR-pMHC affinity, ultimately affecting induction of T-cell proliferation. Here we found that ICAM-1 but not ICAM-2 on APCs is directly involved in the initial *in vitro* activation of MOG-specific T cells.

MOG-reactive T cells differentiate into Th1 and Th17 effector cells that will infiltrate the CNS. We previously reported that endothelial ICAM-1 and ICAM-2 are essential for Th1 cell polarization and crawling on the BBB *in vitro* (14, 16). Epifluorescence IVM confirmed the important role for ICAM-1 and ICAM-2 in supporting sustained Th1 cells-BBB interaction *in vivo* (14). Furthermore, adoptive transfer of Th1 cells into ICAM-1/-2^−/−^ C57BL/6J mice results in ameliorated EAE compared to WT recipients.

Previous reports suggested that LFA-1/ICAM-1 interactions are more important for Th17 than Th1-cell migration across the BBB (17). Therefore, we here compared side-by-side autoaggressive Th1 vs. Th17-cell trafficking across the BBB in the presence or absence of ICAM-1 and ICAM-2. *In vitro* and *in vivo* live cell imaging revealed an intrinsically higher ability of Th1 over Th17 cells to arrest on BBB endothelial cells. As we and others have shown before that α_4_-integrins mediate arrest of Th1 cells to the BBB *in vitro* and *in vivo* (16, 36, 60, 61), it is tempting to speculate that this is due to the higher expression of α_4_ integrins on Th1 vs. Th17 cells, as also reported by other studies (17). We did not observe any differences in the role of endothelial ICAM-1 and ICAM-2 in the post-arrest behavior of Th1 and Th17 cells suggesting that both T cell subsets rely on similar interactions to polarize, probe, crawl on and transmigrate across the BBB. As lack of ICAM-1 and ICAM-2 equally affected the migration of Th1 and Th17 cells across the BBB, we next explored the impact of absence of ICAM-1 and ICAM-2 on the development of Th1 vs. Th17-cell mediated typical and atypical EAE. As observed by others, transfer of encephalitogenic Th1 cells induced typical signs of EAE, while transfer of encephalitogenic Th17 cells induced atypical and typical EAE (44, 62, 63). The ability of Th17 cells to induce atypical EAE has been attributed to preferential migration of Th17 cells into the brain rather than into the spinal cord, in an LFA-1/ICAM-1-dependent manner in Rag1^−/−^ C57BL/6 recipient mice lacking mature adaptive immunity (17). Here we induced Th1 and Th17 cell-mediated tEAE in immune competent WT or ICAM-1/-2^−/−^ C57BL/6J mice and observed the development of typical signs of EAE upon transfer of Th1 cells, while transfer of Th17 cells caused both typical and atypical signs of EAE.

We here observed that absence of ICAM-1 and ICAM-2 resulted in a significant amelioration of both Th1 and Th17-cell mediated EAE, which is in accordance to our *in vitro* and *in vivo* observations that both T-cell subsets require ICAM-1 and ICAM-2 for crossing the BBB. Employing ICAM-1/-2^−/−^ C57BL/6J mice with ubiquitous lack of ICAM-1 and ICAM-2 on all cell types we cannot exclude a role for ICAM-1 and ICAM-2 on cells other than those investigated here in EAE pathogenesis. Thus, inducible tissue specific depletion of ICAM-1 and ICAM-2 would be an interesting future perspective to dissect the precise role of ICAM-1 and ICAM-2 in EAE pathogenesis.

To investigate how lack of ICAM-1 and ICAM-2 affect T-cell infiltration into the brain vs. the spinal cord, we isolated CD45^+^ infiltrating cells from the brains and spinal cords of mice suffering from atypical or typical Th17 cell-mediated EAE. We here confirm that preferential CD4^+^ T cell infiltration into the brain vs. spinal cord is associated with atypical vs. typical EAE, respectively. To further define the CD4^+^ T cell subsets infiltrating the brain and in the spinal cord during EAE, we determined the numbers of CNS infiltrating GM-CSF^+^, IFN-γ^+^ and IL-17^+^ T cells. Irrespective of typical or atypical signs of EAE, the majority of the signature cytokine-producing CD4^+^ T cells were found in the brain rather than in the spinal cord. This suggests that spinal cord infiltration of CD4^+^ T cells is sufficient to induce typical EAE with a mechanism that does not require sustained cytokine expression by the T cells. Initial infiltration of encephalitogenic CD4^+^ T cells into the spinal cord parenchyma may trigger recruitment of monocytes that differentiate into macrophages causing axonal damage and disease progression (49). Importantly, lack of ICAM-1 and ICAM-2 reduced the number of cytokine-producing CD4^+^ T cells able to infiltrate the brain during EAE, probably due to their impaired trafficking across the BBB level. Although our experimental set up does not allow to identify those Th17 cells that were adoptively transferred for inducing EAE, based on our observations it is plausible to conclude that in ICAM-1/-2^−/−^ C57BL/6J mice a lower number of transferred Th17 cells were able to cross the BBB, resulting in a significantly lower number of CD4^+^ IL-17^+^ T cells detected in the CNS of ICAM-1/-2^−/−^ vs. WT C57BL/6J mice suffering from EAE. Importantly, although in a 2-fold lower amount compared to IL-17 and GM-CSF-producing CD4^+^ T cells, we found CD4^+^ IFN-γ^+^ T cells in the brain of mice suffering from Th17-cell mediated EAE. This may be due to the plasticity of the Th17 lineage, which has been observed to assume a Th1-like phenotype, with concomitant or sequential expression of IL-17 and IFN-γ during EAE (64, 65) and in MS patients (66).

In summary, our present study highlights an important role of ICAM-1 on APCs in activating encephalitogenic T cells, which is not replaced by ICAM-2. In contrast, in previous studies we have shown the essential roles of both, endothelial ICAM-1 and ICAM-2 in mediating T-cell polarization and crawling on the BBB endothelium under physiological flow (14, 16). Our present data confirm that endothelial ICAM-1 and ICAM-2 support in a comparable manner post-arrest interaction of Th1 and Th17 cells with the BBB during EAE. Interestingly, lack of ICAM-1 and ICAM-2 rather affects T-cell trafficking into the brain than into the spinal cord, thus differentially affecting atypical and typical signs of EAE. As the choroid plexus was suggested as an alternative Th17 cell entry site into the brain (67), it remains to be shown if ICAM-1 and ICAM-2 are involved in T-cell trafficking into the brain via the choroid plexus. The cell specific involvement of ICAM-1 and ICAM-2 in EAE pathogenesis observed in the present study supports the notion that further studies may allow for therapeutic targeting adhesion molecules to block CNS entry of pathogenic T cells while leaving CNS immune surveillance intact.

## Data Availability Statement

The datasets generated for this study are available on request to the corresponding author.

## Ethics Statement

The animal study was reviewed and approved by the Veterinary office of the Canton Bern.

## Author Contributions

BE conceptualized, supervised, and provided the funding for this research. JS, FS, UD, and CP provided the methodology. NH, LM, FM, CB, DD, HT, and EK performed the experiments and/or provided technical expertise. NH wrote the original draft of the manuscript. LM wrote and edited the second draft of the manuscript. NH and LM assembled the figures and performed the statistical analysis. All authors contributed to the manuscript revision, read, and approved the submitted version.

### Conflict of Interest

The authors declare that the research was conducted in the absence of any commercial or financial relationships that could be construed as a potential conflict of interest.
