# Supplementary material for: Intercellular Adhesion Molecule-1 (ICAM-1) and ICAM-2 Differentially Contribute to Peripheral Activation and CNS Entry of Autoaggressive Th1 and Th17 Cells in Experimental Autoimmune Encephalomyelitis
Source: Front Immunol. 2020 Jan 14;10:3056. doi: 10.3389/fimmu.2019.03056 (PMC6970977; doi:10.3389/fimmu.2019.03056)
Supplement: Supplementary file 7 [file Data_Sheet_1.PDF]

Supplementary Material

Supplementary Video 1. 2P-IVM image sequence of the interaction between a 2D2 GFP $CD4^+$ T cell between WT and ICAM-1/-$2^{-/-}$ DCs.

2P-IVM imaging of the popliteal LN showing the interaction of a $CD4^+$ T cell (green) migrated from an HEV (brown) with a WT DC (blue) and ICAM-1/-2-/- (red) DCs. The interaction with WT DCs is preferred over time. Time is shown in minutes and seconds. Bar, 32 µm.

Supplementary Video 2. 2P-IVM image sequence showing 2D2 GFP $CD4^+$ T cells (green) interacting with WT (blue) and ICAM-1^{null}//ICAM-$2^{-/-}$ (red) DCs pulsed with 100 µg/ml $MOG_{aa35-55}$ peptide.

2P-IVM imaging of the popliteal LN showing the interactions of $CD4^+$ T cells with WT DCs (blue lines) or ICAM-1/-$2^{-/-}$ DCs (red lines). Time is shown in minutes and seconds. Bar, 22 µm.

Supplementary Video 3. Live cell imaging of *in vitro* polarized $CD4^+$ Th1 and Th17 cells interacting with WT pMBMECs.

In vitro live cell imaging of Th1 (red) and Th17 (green) cells migrating on WT pMBMECs. The timer in the top left corner shows minutes:seconds. The arrow in the top right corner shows the direction of the flow. Examples of the most abundant migratory behaviors are shown in the digital zoom-in.

Supplementary Video 4. Live cell imaging of in vitro polarized $CD4^+$ Th1 and Th17 cells interacting with ICAM-1/-$2^{-/-}$ pMBMECs.

In vitro live cell imaging of Th1 (red) and Th17 (green) cells migrating on ICAM-1/-$2^{-/-}$ pMBMECs. The timer in the top left corner shows minutes:seconds. The arrow in the top right corner shows the direction of the flow. Examples of the most abundant migratory behaviors are shown in the digital zoom-in.

Supplementary Video 5. 2P-IVM imaging of the intraluminal crawling of *in vitro* polarized $CD4^+$ Th1 and Th17 cells within cervical spinal cord post-capillary venules in WT C57BL/6J recipient mice with EAE.

In vitro polarized $CD4^+$ Th1 and Th17 cells were systemically injected via carotid artery catheter into a surgically prepared WT C57BL/6J mouse at the onset of EAE on day 12 post-immunization. A x-y-t time-lapse sequence of a 350 µm × 350 µm scan field at a depth of 130 µm and 15-stacks with 4 µm spacing has been acquired. Contrast enhancement of the blood vessels was achieved by inject of Alexa Fluor 633 conjugated anti-endoglin antibody. The video shows Th1 (blue) and Th17 (green) cells intraluminal crawling in cervical spinal cord post-capillary venules with and against the direction of the blood flow. Time is shown in minutes and seconds.

Supplemental Video 6. 2P-IVM imaging of the intraluminal crawling of *in vitro* polarized $CD4^+$ Th1 and Th17 cells within cervical spinal cord post-capillary venules in ICAM-1/-$2^{-/-}$ C57BL/6J recipient mice with EAE.

In vitro polarized $CD4^+$ Th1 and Th17 cells were systemically injected via carotid artery catheter into a surgically prepared ICAM-1/-$2^{-/-}$ C57BL/6J mouse at the onset of EAE on day 11 post-immunization. A x-y-t time-lapse sequence of a 400 µm × 400 µm scan field at a depth of 94 µm and 10-stacks with 4 µm spacing has been acquired. Contrast enhancement of the blood vessels was achieved by inject of Alexa Fluor 594 conjugated anti-endoglin antibody. The video shows Th1 (green) and Th17 (blue) cells interaction with cervical spinal cord post-capillary venules, characterized by detachment or impaired crawling. Time is shown in minutes and seconds.
