# Supplementary material for: Intercellular Adhesion Molecule-1 (ICAM-1) and ICAM-2 Differentially Contribute to Peripheral Activation and CNS Entry of Autoaggressive Th1 and Th17 Cells in Experimental Autoimmune Encephalomyelitis
Source: Front Immunol. 2020 Jan 14;10:3056. doi: 10.3389/fimmu.2019.03056 (PMC6970977; doi:10.3389/fimmu.2019.03056)
Supplement: Supplementary file 8 [file Image_1.PDF]

Supplementary Material

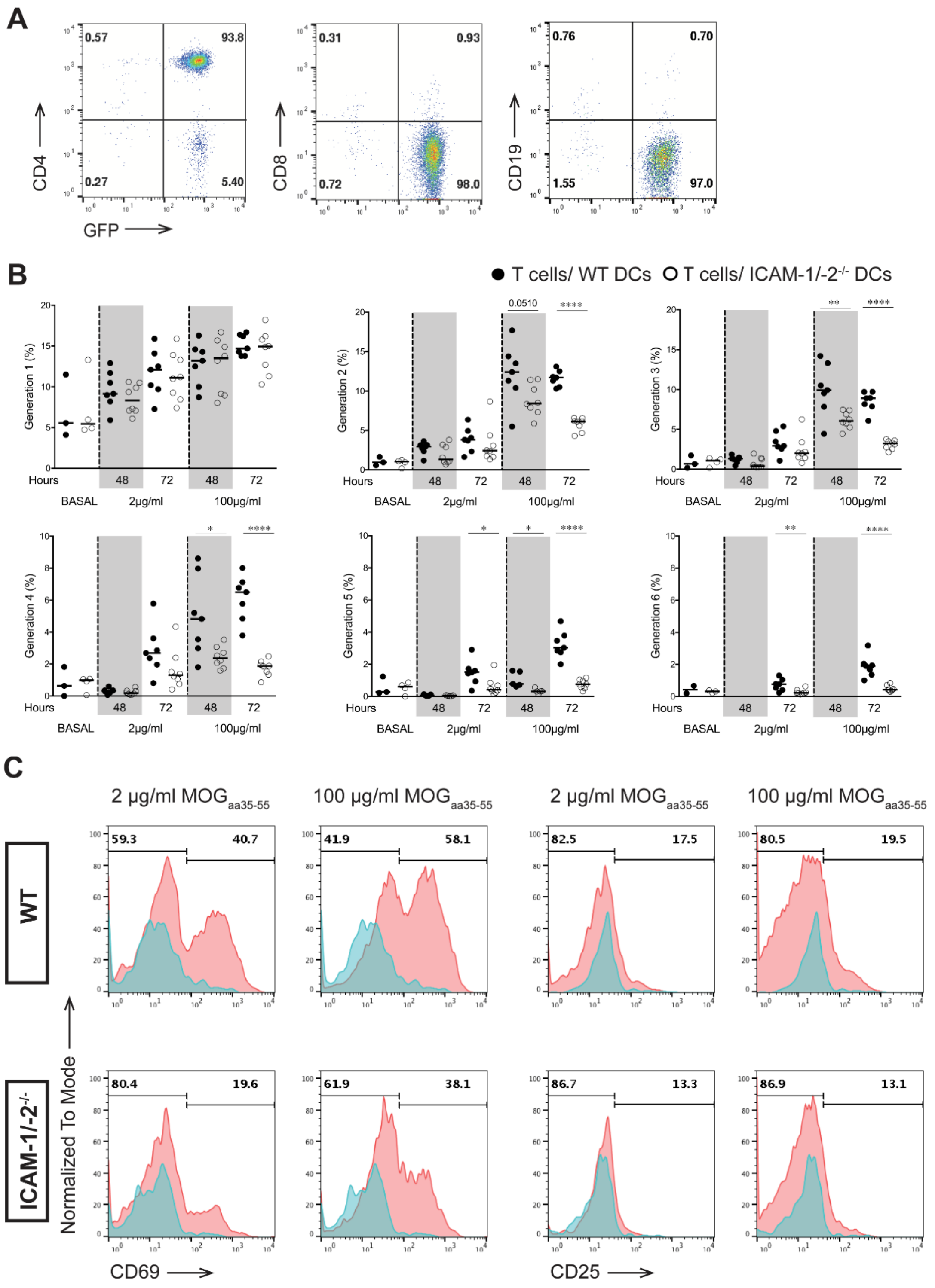

Supplementary Figure 1. Purity of 2D2 GFP$^+$ T cells and quantification of individual generations of divided 2D2 GFP$^+$ CD4$^+$ T cells stimulated with WT or ICAM-1/-2$^{-/-}$ DCs and representative flow cytometry plots of CD69 and CD25 expression by divided 2D2 CD4$^+$ T cells proliferated in recipient mice.

(A) Spleen and PLNs of 2D2 GFP$^+$ C57BL/6J mice were collected, and single cell suspensions of these organs were pooled. The purity of CD4$^+$ T cells was examined by flow cytometry and staining for CD4, CD8 and CD19. The results shown are one representative out of 4 independent experiments. **(B)** Percentages of individual generations of divided 2D2 CD4$^+$ T cells stimulated with WT or ICAM-1/-2$^{-/-}$ DCs pulsed with 2 µg/ml, 100 µg/ml, or no (BASAL) $MOG_{aa35–55}$ peptide. Cell divisions were monitored via dilution of the e670 fluorescent dye 48 h and 72 h after transfer of e670 labeled 2D2 GFP CD4$^+$ T cells into recipient mice. Each dot represents PLN T cells pooled from one mouse. Data were pooled from 2 individual experiments with a total of 7-8 mice at each time point and 3-4 mice at BASAL level and shown as mean. Data were analyzed using repeated measure ANOVA with Bonferroni post-test. *$p < 0.05$, ** $p < 0.01$, **** $p < 0.0001$. **(C)** Representative flow cytometry plots showing the percentages of CD69 and CD25 expression by divided 2D2 CD4$^+$ T cells, 48 h after transfer of e670 labeled 2D2 GFP CD4$^+$ T cells into recipient mice containing WT or ICAM-1/-2$^{-/-}$ DCs pulsed with 2 µg/ml (red), 100 µg/ml (red) or no (blue) $MOG_{aa35–55}$ peptide.
