# Supplementary material for: Intercellular Adhesion Molecule-1 (ICAM-1) and ICAM-2 Differentially Contribute to Peripheral Activation and CNS Entry of Autoaggressive Th1 and Th17 Cells in Experimental Autoimmune Encephalomyelitis
Source: Front Immunol. 2020 Jan 14;10:3056. doi: 10.3389/fimmu.2019.03056 (PMC6970977; doi:10.3389/fimmu.2019.03056)
Supplement: Supplementary file 9 [file Image_2.PDF]

Supplementary Material

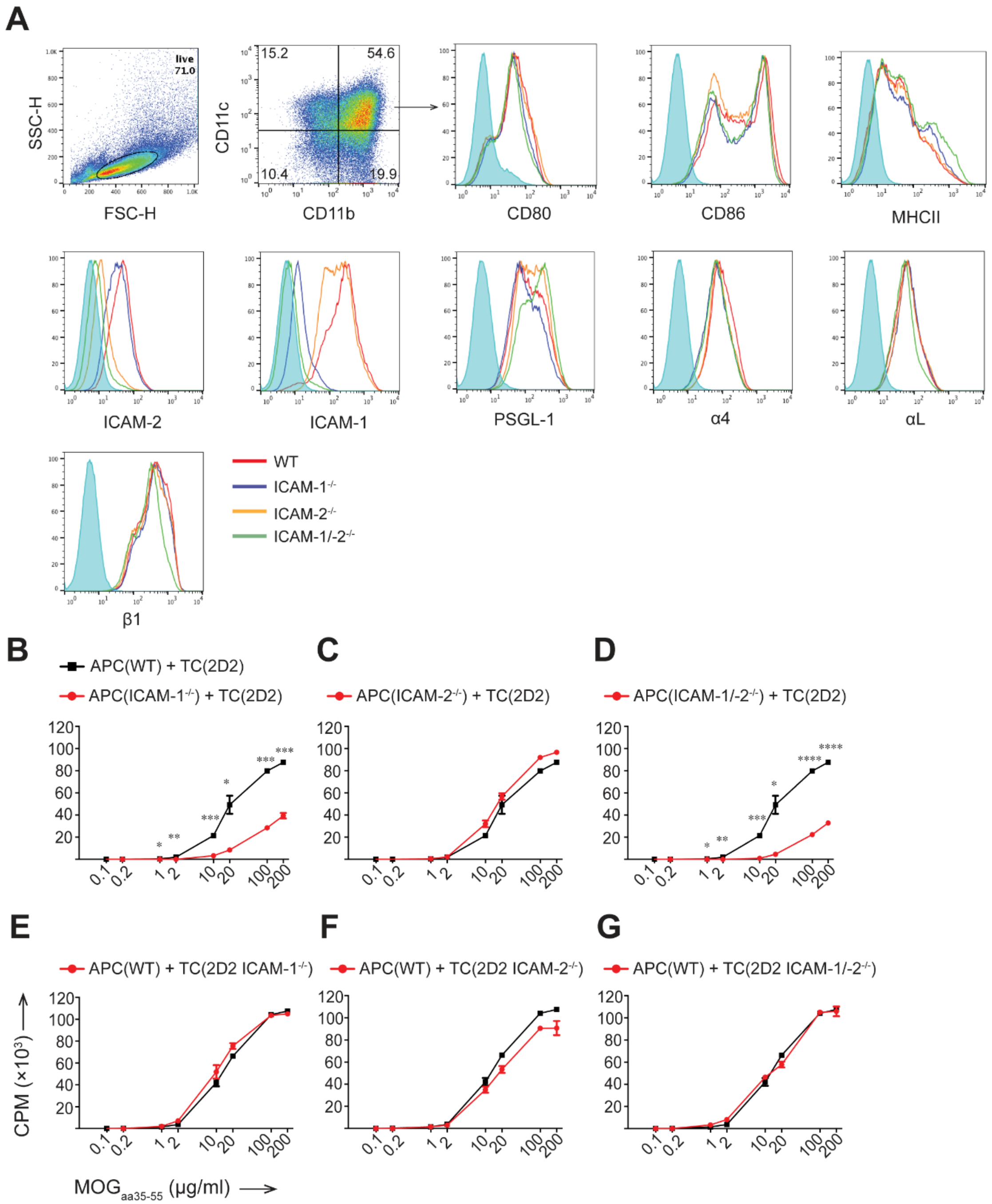

Supplementary Figure 2. Evaluation of DCs phenotype harvested from WT, ICAM-1$^{-/-}$, ICAM-2$^{-/-}$ and ICAM-1/-2$^{-/-}$ C57BL/6J mice and decreased *in vitro* antigen-specific CD4^{+} T-cell

proliferation in the absence of ICAM-1 but not ICAM-2 on splenocytes as antigen presenting cells.

(A) LPS-stimulated DCs were analyzed by flow cytometry using anti-CD11b and anti-CD11c antibodies. Expression of CD80, CD86, MHC class II, ICAM-2, ICAM-1, PSGL-1, α_4, α_L and β_1 integrins were compared on LPS-activated $CD11b^+CD11c^+$ DCs harvested from WT (red line), ICAM-$1^{-/-}$ (blue line), ICAM-$2^{-/-}$ (orange line), and ICAM-1/-$2^{-/-}$ (green line) C57BL/6J mice. Histogram with the cyan color displays the respective isotype Ig. Three individual experiments were performed. **(B-G)** Purified $CD4^+$ T cells (TC) were co-cultured with irradiated mixed splenic APCs (APC) with a ratio of 5:1 APCs/TCs. Different concentrations of $MOG_{aa35-55}$ peptide were added to the co-cultures and incubated for 72 hours before the cells were pulsed with [^{3}H]-thymidine. Data shown are representative of 3 individual experiments per condition. Proliferation is displayed as ^{3}H-thymidine incorporation counts per minute (CPM) per well. Presented results are the mean ± SEM after subtraction of background proliferation, which was determined in the absence of antigen ($MOG_{aa35-55}$ peptide). Statistical differences between the groups were calculated with the paired Student's *t*-test. $*p < 0.05$, $**p < 0.01$, $***p < 0.001$, $****p < 0.0001$. **(B-D)** TCs from 2D2 C57BL/6J mice co-cultured with irradiated APCs from WT, ICAM-$1^{-/-}$, ICAM-$2^{-/-}$ or ICAM-1/-$2^{-/-}$ C57BL/6J mice. **(E-G)** TCs from 2D2, 2D2 ICAM-$1^{-/-}$, 2D2 ICAM-$2^{-/-}$ or 2D2 ICAM-1/-$2^{-/-}$ C57BL/6J mice co-cultured with irradiated APCs from WT C57BL/6J mice.
