# Supplementary material for: Intercellular Adhesion Molecule-1 (ICAM-1) and ICAM-2 Differentially Contribute to Peripheral Activation and CNS Entry of Autoaggressive Th1 and Th17 Cells in Experimental Autoimmune Encephalomyelitis
Source: Front Immunol. 2020 Jan 14;10:3056. doi: 10.3389/fimmu.2019.03056 (PMC6970977; doi:10.3389/fimmu.2019.03056)
Supplement: Supplementary file 10 [file Image_3.PDF]

Supplementary Material

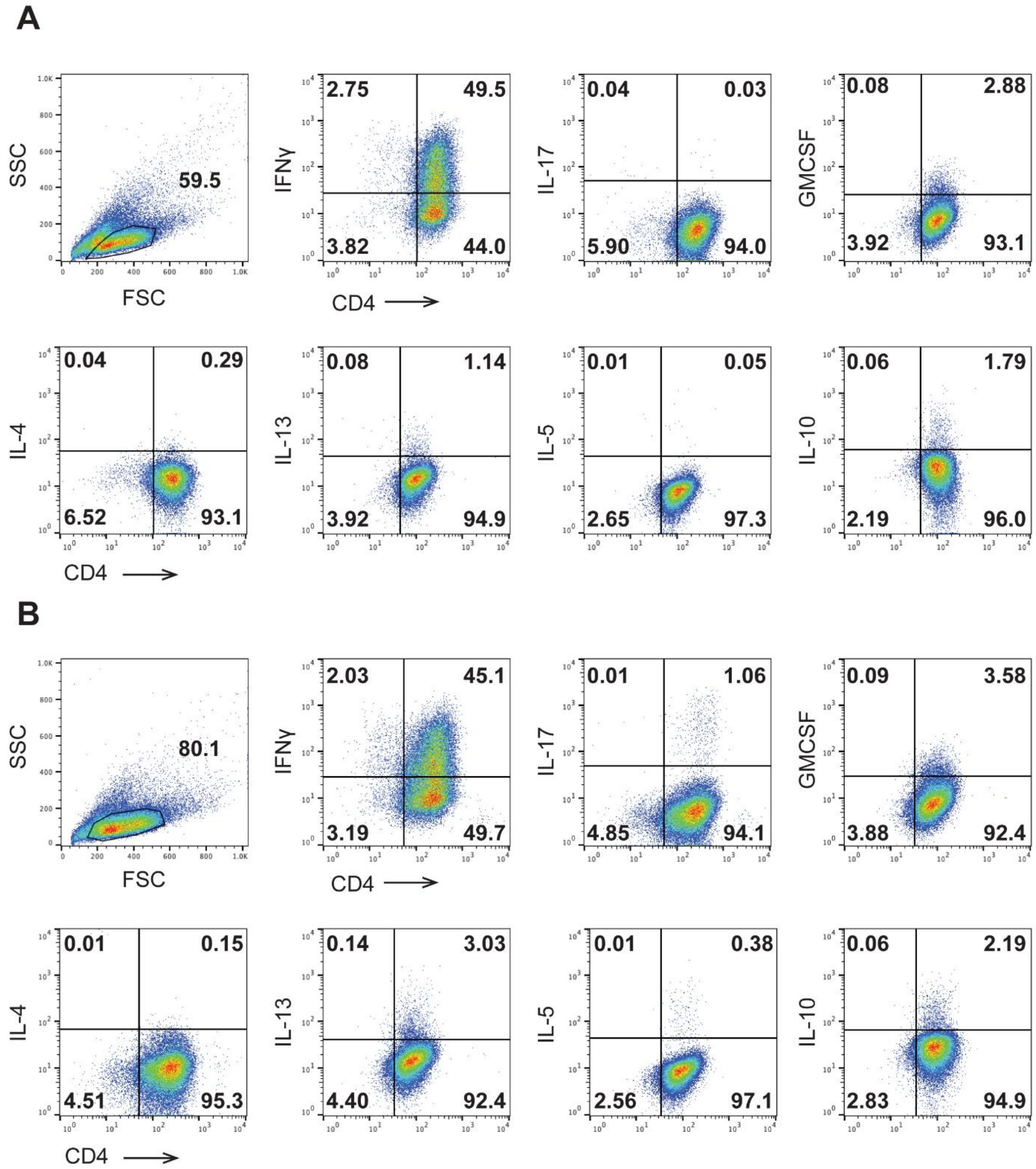

Supplementary Figure 3. Cytokine profile of 2D2 and 2D2 ICAM-1/-2$^{-/-}$ CD4^{+} T cells stimulated with splenic APCs.

(A and B) Purified $CD4^+$ T cells from either 2D2 **(A)** or 2D2 ICAM-1/-$2^{-/-}$ **(B)** were co-cultured with irradiated splenic APCs from WT C57BL/6J mice and stimulated with $MOG_{aa35\text{-}55}$ peptide (40 µg/ml) for 72 hours. The cytokine repertoire of activated T cells after 5 hours stimulation with PMA/Ionomycin was analyzed by flow cytometry. Two independent experiments were done per condition.
