# Supplementary material for: Intercellular Adhesion Molecule-1 (ICAM-1) and ICAM-2 Differentially Contribute to Peripheral Activation and CNS Entry of Autoaggressive Th1 and Th17 Cells in Experimental Autoimmune Encephalomyelitis
Source: Front Immunol. 2020 Jan 14;10:3056. doi: 10.3389/fimmu.2019.03056 (PMC6970977; doi:10.3389/fimmu.2019.03056)
Supplement: Supplementary file 11 [file Image_4.PDF]

Supplementary Material

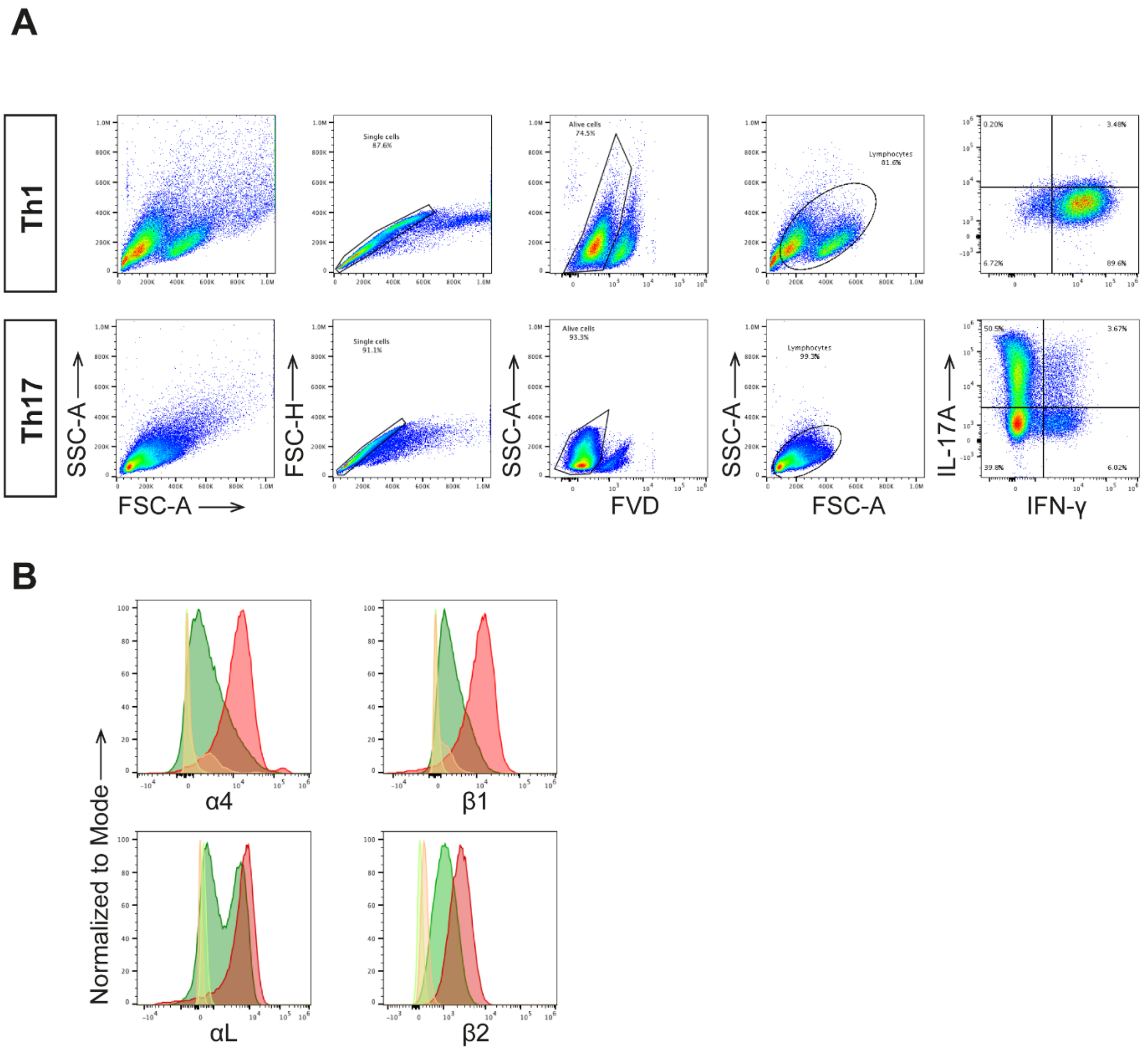

Supplementary Figure 4. Cytokine profile and surface molecules expression of *in vitro* polarized Th1 and Th17 cells.

(A and B) Purified CD4$^+$ T cells harvested from 2D2 C57BL/6J mice were polarized towards Th1 subset (with IL-12 and IL-2) and Th17 subset (with IL-6, TGF-β1, IL-1β and IL-23) and cultured for

5 day. Representative plots from three independent experiments. **(A)** After 5 days of *in vitro* activation and polarization, IFN-γ and IL-17 production were assessed via flow cytometry as the hallmark cytokine of Th1 and Th17 subsets, respectively. Gating strategy has been selected to exclude duplets and dead cells, using Fixable Viability Dyes (FVD). **(B)** Surface molecules expression of α4, β1, αL and β2 integrins was evaluated on Th1 cells (dark red histogram) and Th17 cells (dark green histogram) via flow cytometry. Light red and light green histograms show the respective isotype Ig of Th1 cells and Th17 cells.
