# Supplementary material for: Intercellular Adhesion Molecule-1 (ICAM-1) and ICAM-2 Differentially Contribute to Peripheral Activation and CNS Entry of Autoaggressive Th1 and Th17 Cells in Experimental Autoimmune Encephalomyelitis
Source: Front Immunol. 2020 Jan 14;10:3056. doi: 10.3389/fimmu.2019.03056 (PMC6970977; doi:10.3389/fimmu.2019.03056)
Supplement: Supplementary file 12 [file Image_5.PDF]

Supplementary Material

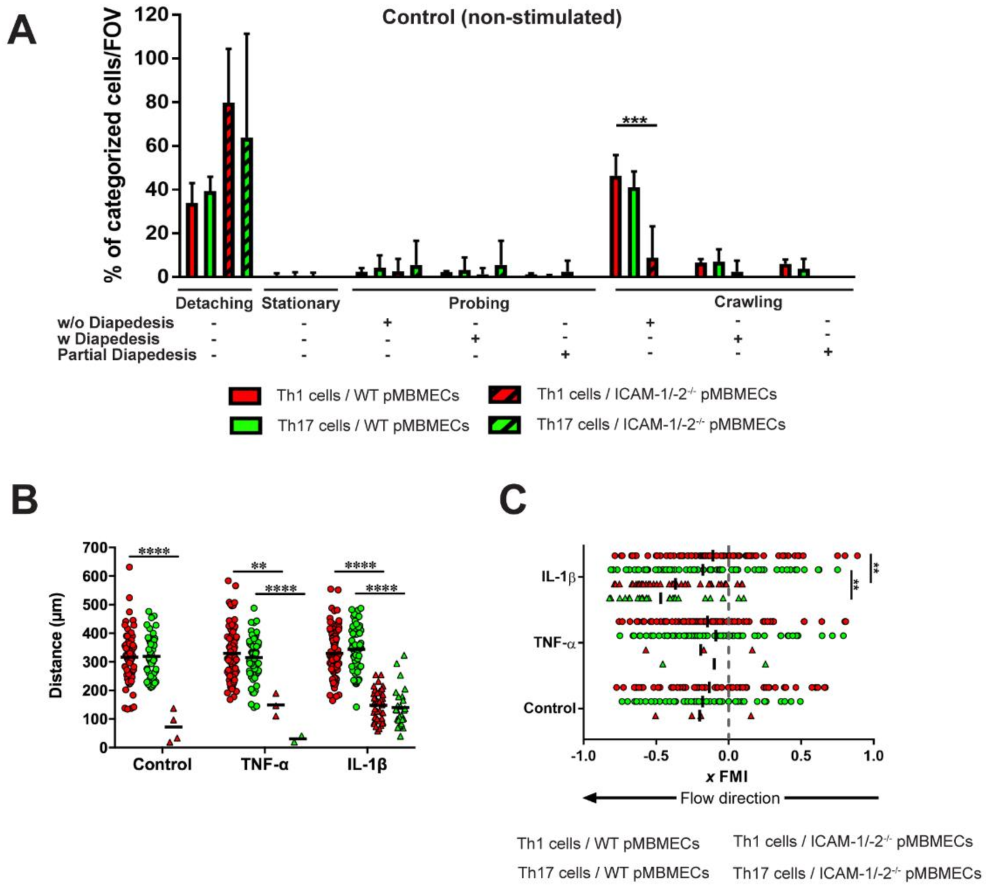

Supplementary Figure 5. Live cell imaging analysis of the migratory behavior of *in vitro* polarized Th1 and Th17 CD4$^+$ cells with WT or ICAM-1/-2$^{-/-}$ pMBMECs.

(A) Post arrest dynamic behavior during 30 minutes of recording time of *in vitro* polarized CD4$^+$ Th1 (red) and Th17 (green) cells on non-stimulated WT (filled bars) and ICAM-1/-2$^{-/-}$ (striped bars) pMBMECs. The behavioral categories are presented as percentage of categorized Th1 and Th17 cells per each condition of pMBMECs. Data are shown are mean ± SD from four independent experiment. **(B and C)** Analysis of CD4$^+$ Th1 (red) and Th17 (green) cells on WT (squares) and ICAM-1/-2$^{-/-}$

(triangles) control, TNFα or IL-1β-stimulated pMBMECs. Each data point represents one crawling track of one Th1 or Th17 cells. Values are shown as mean (Th1 or Th17 crawling tracks on WT pMBMECs ≥ 50; Th1 or Th17 crawling tracks on ICAM-1/-2$^{-/-}$ pMBMECs ≤ 37) and are pooled from 4 individual experiments. Data were analyzed using repeated measure ANOVA with Tukey post-test *$p < 0.05$, ** $p < 0.01$, *** $p < 0.001$, **** $p < 0.0001$. **(B)** Accumulated crawling distance in micrometer (μm) of Th1and Th17 cells on WT and ICAM-1/-2$^{-/-}$ control, TNFα or IL-1β-stimulated pMBMECs. **(C)** *X*-axis Crawling Forward Migration Index (FMI) of Th1 and Th17 cells on WT and ICAM-1/-2$^{-/-}$ control, TNFα or IL-1β-stimulated pMBMECs. Direction of flow is indicated by the arrow.
