# Supplementary material for: Intercellular Adhesion Molecule-1 (ICAM-1) and ICAM-2 Differentially Contribute to Peripheral Activation and CNS Entry of Autoaggressive Th1 and Th17 Cells in Experimental Autoimmune Encephalomyelitis
Source: Front Immunol. 2020 Jan 14;10:3056. doi: 10.3389/fimmu.2019.03056 (PMC6970977; doi:10.3389/fimmu.2019.03056)
Supplement: Supplementary file 13 [file Image_6.pdf]

Supplementary Material

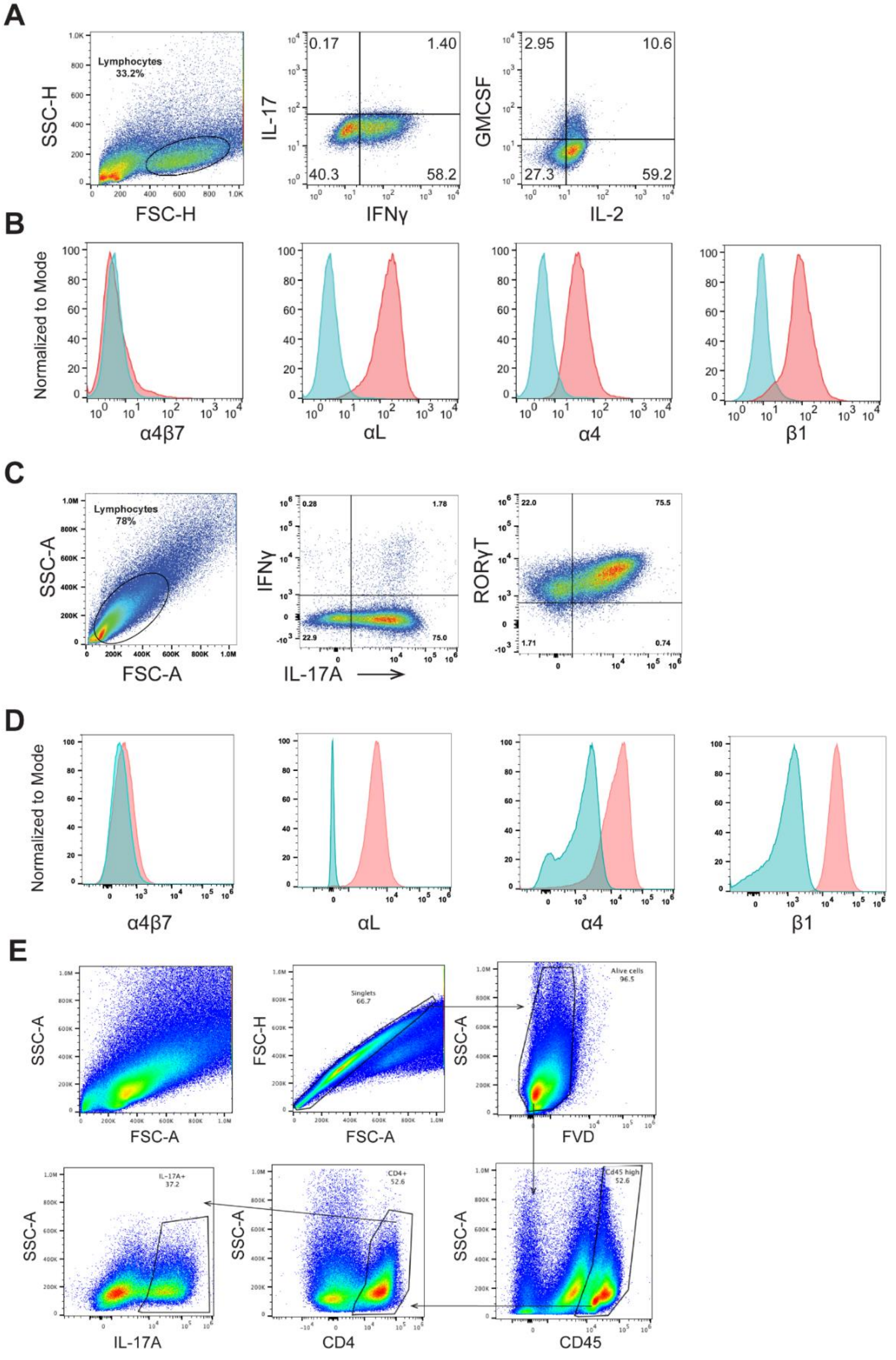

Supplementary Figure 6. Flow cytometry analysis of *in vitro* polarized Th1 and Th17 cells used for induction of Th1 and Th17 cell-mediated EAE and flow cytometry gating strategy of CD45$^+$ infiltrated cells in the CNS.

(A and B) Purified CD4$^+$ T cells harvested from 2D2 C57BL/6J mice were polarized towards Th1 subset (with IL-12 and IL-2) cultured for 9 days. Representative plots from 3 independent experiments. **(A)** Polarization of Th1 cells after 9 days culture was assessed by flow cytometry via production of IFN-γ as hallmark cytokine of Th1 cells. Production of IL-17, IL-2 and GMCSF was analyzed to quantify Th1 subset purity. **(B)** Surface molecules expression of $\alpha_4\beta_7$, α_L, α_4 and β_1 integrins was evaluated on Th1 cells (red histogram) and the respective isotype Ig (blue histogram) after 9 days of culture. **(C and D)** Purified CD4$^+$ T cells harvested from 2D2 C57BL/6J mice were polarized towards Th17 subset (with IL-6, TGFβ1, anti- IFN-γ and anti-IL-4) cultured for 8 days. Representative plots of three independent experiments. **(C)** Polarization of Th17 cells was assessed by flow cytometry via production of IL-17 as signature cytokine of Th17 cells, together with RORγt as signature transcription factor. IFN-γ production was assessed to quantity Th17 subset purity. **(D)** Surface molecules expression of $\alpha_4\beta_7$, α_L, α_4 and β_1 integrins was evaluated on Th17 cells (red histogram) and the respective isotype Ig was shown in each plot (blue histogram) after 8 days of culture. **(E)** Representative plots of the gating strategy used in the flow cytometry analysis of CD45$^+$ infiltrated cells in the brain and spinal cord of WT and ICAM-1/-2$^{-/-}$ mice suffering from Th17 tEAE. Exclusion of dead cells was performed using Fixable Viability Dye (FVD).
