# Supplementary material for: Intercellular Adhesion Molecule-1 (ICAM-1) and ICAM-2 Differentially Contribute to Peripheral Activation and CNS Entry of Autoaggressive Th1 and Th17 Cells in Experimental Autoimmune Encephalomyelitis
Source: Front Immunol. 2020 Jan 14;10:3056. doi: 10.3389/fimmu.2019.03056 (PMC6970977; doi:10.3389/fimmu.2019.03056)
Supplement: Supplementary file 14 [file Image_7.PDF]

Supplementary Material

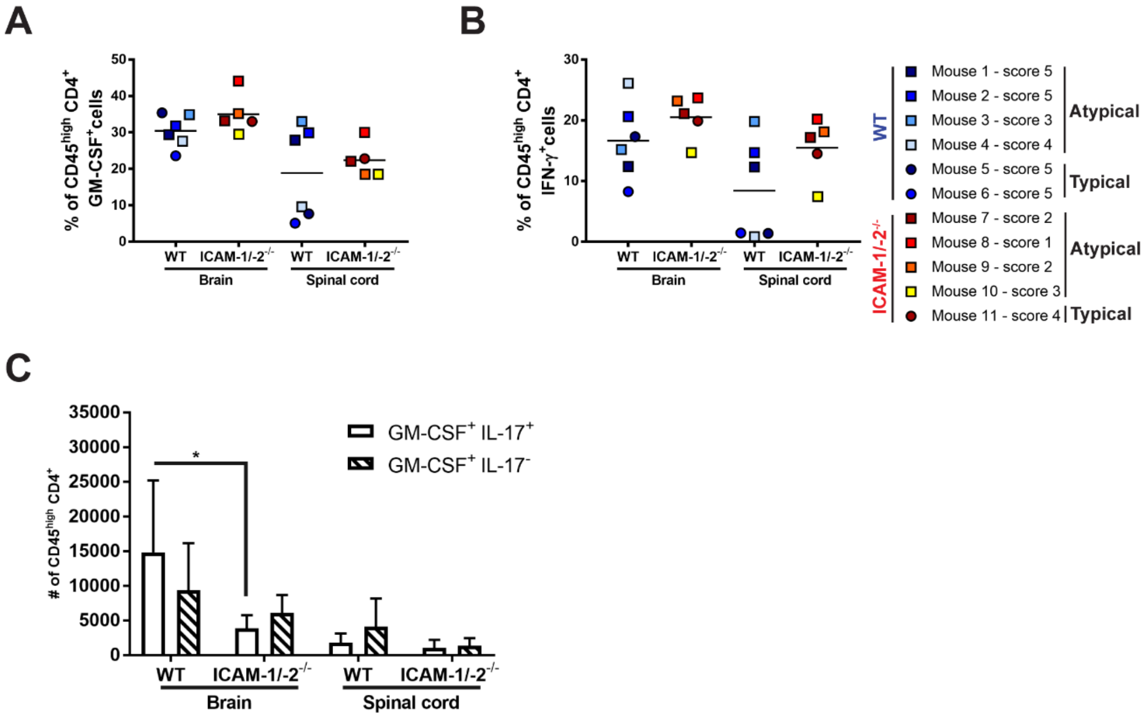

Supplementary Figure 7. Flow cytometry analysis of CD45^{+} CNS infiltrating cells in WT and ICAM-1/-2$^{-/-}$ mice during Th17 cell-mediated EAE.

(A and B) Quantification of the percentage of CD45^{high}CD4^{+}GM-CSF^{+} **(A)** and CD45^{high}CD4^{+}IFN-γ^{+} **(B)** T cells evaluated by flow cytometry analysis in brains and spinal cords of WT (blue shades) and ICAM-1/-2$^{-/-}$ (red shades) recipient mice suffering from typical (circles) or atypical (squares) EAE. Each dot represents one individual mouse and a total of 6 WT and 5 ICAM-1/-2$^{-/-}$ mice were analyzed per each group at the peak of disease (day 16-20 post transfer of *in vitro* polarized CD4^{+} Th17 cells). Data are pooled from 2 individual experiments and shown as mean. Data analyzed using repeated measure two-way ANOVA with Tukey post-test *$p < 0.05$, ** $p < 0.01$. **(C)** Quantification of the numbers of CD45^{high}CD4^{+}GM-CSF^{+}IL-17^{+} and CD45^{high}CD4^{+}GM-CSF^{+}IL-17^{-} T cells evaluated by

flow cytometry analysis in brains and spinal cords of WT and ICAM-1/-2$^{-/-}$ recipient mice suffering from typical and atypical EAE for a total of 6 WT and 5 ICAM-1/-2$^{-/-}$ mice analyzed per each group at the peak of disease (day 16-20 post transfer of *in vitro* polarized CD4^{+} Th17 cells). Data are pooled from 2 individual experiments and shown as mean ± SD. Data analyzed using repeated measure two-way ANOVA with Tukey post-test *$p < 0.05$, ** $p < 0.01$.
